# Anlotinib enhances the antitumor immunity of radiotherapy by activating cGAS/STING in non-small cell lung cancer

**DOI:** 10.1038/s41420-022-01256-2

**Published:** 2022-11-28

**Authors:** Dong Han, Jiajia Zhang, Yawei Bao, Lei Liu, Ping Wang, Dong Qian

**Affiliations:** 1 Department of Radiation Oncology, Tianjin Medical University Cancer Institute and Hospital, National Clinical Research Center for Cancer, Tianjin’s Clinical Research Center for Cancer, Key Laboratory of Cancer Immunology and Biotherapy, 300060 Tianjin, China; 2 Department of Radiation Oncology, YanTai Yuhuangding Hospital, 264000 Yantai, Shandong China; 3 Department of Radiation Oncology, the First Affiliated Hospital of USTC, Division of Life Sciences and Medicine, University of Science and Technology of China, 230001 Hefei, Anhui P.R. China; 4 Department of Radiation Oncology, the First Affiliated Hospital of USTC, 230001 Hefei, Anhui P.R. China

**Keywords:** Non-small-cell lung cancer, Cancer microenvironment, Radiotherapy, Tumour immunology

## Abstract

Radiation resistance and unsatisfactory efficacy of radioimmunotherapy are important barriers to non-small cell lung cancer (NSCLC) treatment. The impacts of anlotinib on radiation and tumor immune microenvironment (TIME) in NSCLC remain to be resolved. Here, we find anlotinib enhances radiosensitivity, and further increases radiotherapy-stimulated CD8^+^ T cell infiltration and activation via triggering cGAS/STING pathway. Moreover, anlotinib shows significant effects on radioimmunotherapy (radiotherapy plus anti-PD-L1). The addition of anlotinib alleviates CD8^+^ T cell exhaustion, promotes the cytotoxicity and proliferation of CD8^+^ T cells, and boosts immune memory activation. Our work reveals the crucial role of anlotinib in antitumor immunity, and provides preclinical evidence for the application of anlotinib combined with radioimmunotherapy in NSCLC treatment.

## Introduction

Lung cancer is the most commonly diagnosed cancer (11.6%) in men and women and remains the primary cause of cancer-related deaths worldwide [1]. Non-small cell lung cancer (NSCLC) accounts for about 85% of all lung cancer cases [2]. Radiotherapy (RT) plays a significant role in NSCLC treatment. However, radiation resistance and high local failure rate are still the major limitations regarding patient prognosis [3, 4]. The development of immune-checkpoint blockade (ICB) treatment makes RT combined with ICB a promising treatment option, but the synergistic effect remains clinically unsatisfactory [5–7]. Therefore, finding safe combination strategies to enhance the antitumor efficacy of radiation is an urgent requirement.

Typically, ionizing radiation (IR) induces DNA double-strand breaks (DSBs) that directly kills tumor cells. Recently, an increasing number of studies demonstrate that radiation induces double-strand DNA (dsDNA) production and activates cGAS/STING pathway, thereby stimulating innate immunity [8–10]. In addition, RT has opposing effects on the tumor immune microenvironment (TIME). For example, radiation triggers the DNA damage response (DDR) pathway, upregulates the expression of PD-L1 and forms a suppressive TIME, which leads to RT resistance [11, 12].

Anlotinib is a small molecule multitargeted tyrosine kinase inhibitor that exerts a broad inhibitory effect on tumor angiogenesis and growth [13, 14]. Some studies have demonstrated that antiangiogenic agents enhance radiosensitivity and improve the efficacy of RT and immunotherapy [15, 16]. Moreover, antiangiogenic treatment can normalize and reshape the aberrant tumor vasculature, thus alleviating immunosuppression [17, 18]. How anlotinib affects radiosensitivity and radioimmunotherapy is yet to be clarified.

In the present study, we discovered that the addition of anlotinib enhanced radiosensitivity and facilitated the recruitment and activation of CD8^+^ T cells. Also, the role of anlotinib in immune-editing and synergetic treatment with RT and radioimmunotherapy was identified via activation of the cGAS/STING pathway in NSCLC.

## Results

### Anlotinib enhances radiosensitivity of NSCLC in vitro and in vivo

**Fig. 1:**
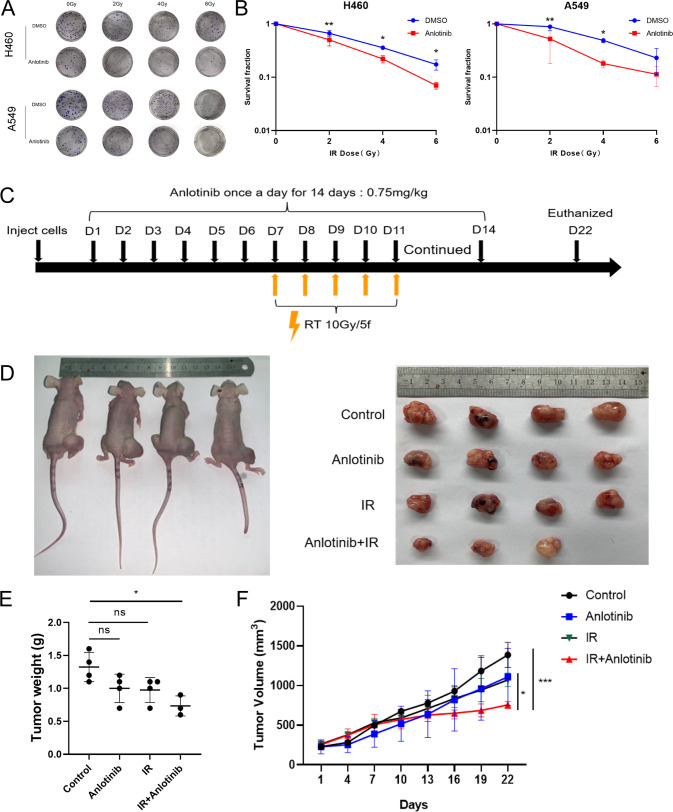
Anlotinib enhances radiosensitivity of NSCLC in vitro and in vivo. **A** Representative images of colony formation of H460 and A549 cells irradiated with 0, 2, 4 and 6 Gy. **B** The cell survival curves were fitted with the linear-quadratic model. **C** When the tumor grew to a volume of approximately 200 mm^3^, mice were randomly divided into the following four groups: control (*n* = 4), anlotinib (*n* = 4), IR (*n* = 4), and IR plus anlotinib (*n* = 3). Experimental schedule of anlotinib and IR combination treatment for H460 tumor-bearing mice. **D**–**F** Tumor weight and volume of xenografts were shown in the graphs. One-way ANOVA with Tukey’s multiple comparison was performed for tumor weight comparison and two-way ANOVA with Tukey’s multiple comparison was conducted for tumor volume analysis with GraphPad Prism 8.0.2. Data are presented as the mean ± standard deviation, ns = not significant, *p < 0.05, **p < 0.01, ***p < 0.001.

To examine the impact of anlotinib on the growth of NSCLC cells, H460 and A549 cells were incubated with different concentrations of anlotinib for 24 h, 48 h, and 72 h, and the cell viability was measured using the CCK-8 assay. The results revealed that anlotinib suppresses cell viability in a dose- and time-dependent manner (Supplementary Fig. 1). Clonogenic assay showed that pretreatment with anlotinib followed by IR significantly inhibited the colony formation of NSCLC cells after normalization of survival at 0 Gy for different pretreatment groups compared to IR alone (Fig. 1A, B). Next, we investigated the effect of anlotinib on radiosensitivity in vivo. The experimental schedule was illustrated in Fig. 1C. Similarly, we found that anlotinib combined with IR remarkably delayed tumor growth compared to IR alone in tumor-bearing nude mice (Fig. 1D–F). In addition, no distinct pathological changes were observed in the vital organs of mice throughout the experimental period (Supplementary Fig. 2). Collectively, these data demonstrate that anlotinib sensitize NSCLC to irradiation in vitro and in vivo.

### Anlotinib inhibits repair of IR-induced DNA DSBs and stimulates the STING pathway

**Fig. 2:**
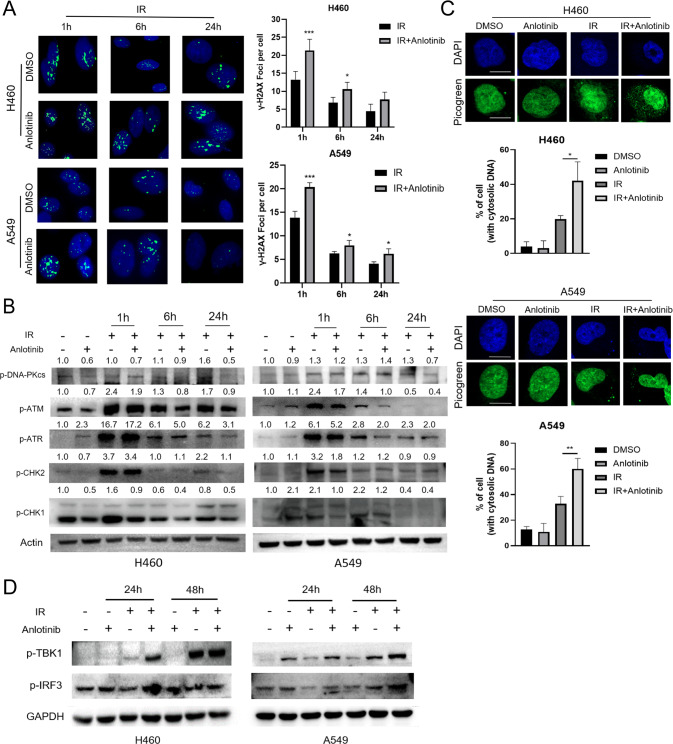
Anlotinib inhibits repair of IR-induced DNA double-strand breaks and enhances activation of STING pathway. **A** Detection of γ-H2AX foci was performed at the indicated time point after irradiation (4 Gy). Data are expressed as the mean ± standard deviation. Student’s *t* test. **B** Expression levels of phosphorylated DNA-PKcs, ATM, ATR, CHK2, and CHK1 were detected at the indicated time point after irradiation (4 Gy). Western blot quantification of phosphorylated DNA-PKcs, ATM, ATR, CHK2, and CHK1, normalized to Actin. Results are shown as a representative of *n* = 3 independent repeats. **C** Representative images and quantitative analysis of PicoGreen staining. Cells with cytosolic DNA were counted. Scale bar, 10 μm. Student’s *t* test. **D** Western blot revealed that anlotinib combined with IR increased the expression of p-TBK1 and p-IRF3. *p < 0.05, **p < 0.01, ***p < 0.001.

The most lethal DNA lesions induced by irradiation are DSBs [19]. Next, we investigated whether anlotinib pretreatment increased IR-induced DNA damage and impaired DNA DSB repair. After exposure to IR, more foci formation of γ-H2AX was observed in the combination group compared to the irradiation alone group in both H460 and A549 cells at 1 h (Fig. 2A). A large number of residual γ-H2AX foci remained in the combination groups compared to the irradiation alone groups at 24 h after IR, indicating unrepaired and potentially lethal DSBs (Fig. 2A). As an important protein for DSB repair, 53BP1 is recruited to break sites and involves in non-homologous end joining [20]. The number of 53BP1 foci increased markedly after exposure to IR compared to the unirradiated cells, and abundant foci were observed in combination groups (Supplementary Fig. 3). Moreover, we evaluated the impact of anlotinib on the critical molecules for DSBs repair: DNA-PKcs, ATM, ATR, CHK2, and CHK1. Compared to IR alone, anlotinib pretreatment followed by IR decreased the expression of phosphorylated DNA-PKcs, ATM, ATR, CHK2, and CHK1 (Fig. 2B).

Deficient DSB repair leads to the accumulation of impaired DNA, which leaks into the cytoplasm and triggers cGAS, the cytosolic DNA sensor, and then activates STING and its downstream proteins [21, 22]. As expected, anlotinib induced the accumulation of cytosolic dsDNA upon IR exposure (Fig. 2C). Furthermore, the expression levels of phosphorylated TBK1 and IRF3, two crucial factors in the STING pathway, were increased in IR plus anlotinib treatment (Fig. 2D). To confirm whether the activation of STING pathway was specific, we investigated the expression levels of cGAS and STING in different NSCLC cell lines. Results revealed that the expression levels of cGAS and STING varied a lot between different cell lines, and their levels were lower in A549 and H460 cell lines (Supplementary Fig. 4). Collectively, these results suggest that anlotinib hinders IR-induced DNA DSB repair, increases the accumulation of cytosolic dsDNA, and enhances the activation of STING pathway as a response to IR treatment.

### Anlotinib combined with IR enhances CD8^+^ T cell infiltration

**Fig. 3:**
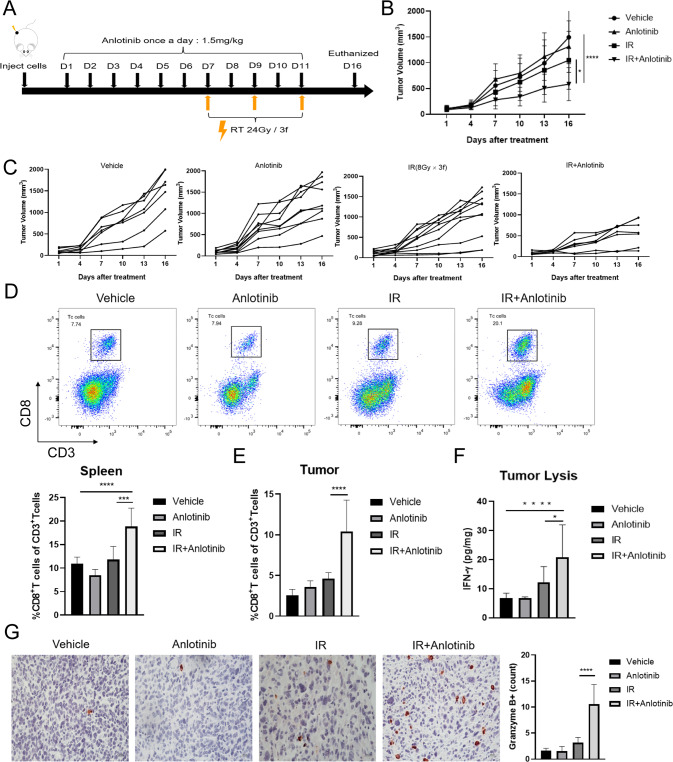
Anlotinib combined with IR increases CD8^+^ T cell recruitment. **A** Schematic illustration of treatment schedule for LLC-OVA tumor-bearing mice. **B** When the tumor volume reached an average of 100 mm^3^, the mice were randomly divided into different treatment groups, and tumor volume was measured every 3 days. Two-way ANOVA with Tukey’s multiple comparison. **C** Individual growth curves for the subcutaneous tumor in each group (Vehicle, *n* = 7; Anlotinib, *n* = 9; IR, *n* = 10; IR + Anlotinib, *n* = 7). **D** Representative flow cytometry staining (upper panel) and quantification of percentage (lower panel) of CD8^+^ T cells in spleen on day 16 after treatment initiation (Vehicle, *n* = 8; Anlotinib, *n* = 8; IR, *n* = 5; IR + Anlotinib, *n* = 7). One-way ANOVA with Tukey’s multiple comparison. **E** Percentage of CD8^+^ T cells in tumor on day 16 after treatment initiation (Vehicle, *n* = 9; Anlotinib, *n* = 8; IR, *n* = 6; IR + Anlotinib, *n* = 7). One-way ANOVA with Tukey’s multiple comparison. **F** Intratumoral IFN-γ concentrations were measured using Elisa. Relative IFN-γ level was calculated by normalizing to protein concentration of each sample. One-way ANOVA with Tukey’s multiple comparison. **G** Representative immunostaining images and quantification of GzmB in subcutaneous tumors were assessed with IHC. Scale bar, 200 μm. One-way ANOVA with Tukey’s multiple comparison. Data are presented as the mean ± standard deviation, *p < 0.05, **p < 0.01, ***p < 0.001, ****p < 0.0001.

Based on the above results, it could be speculated that anlotinib may play a role in regulating immune microenvironment. Next, we performed the following treatments in LLC-OVA tumor-bearing mice (Fig. 3A). We found that anlotinib combined with IR significantly inhibited tumor growth (Fig. 3B, C; mean tumor volume ± SEM at day 16: 1049 ± 178.5 mm^3^ IR vs. 584.9 ± 120.4 mm^3^ IR plus anlotinib, *p* = 0.0195). Typically, the addition of anlotinib to IR remarkably increased the percentage of CD8^+^ T cells compared to IR alone in both spleens and tumors (Fig. 3D, E). Moreover, the combination of IR and anlotinib significantly increased the expression of interferon-gamma (IFN-γ) (Fig. 3F) and GzmB (Fig. 3G), a cytotoxic granule reflective of CD8^+^ T cell effector function. Together, these results indicate that the combination treatment improves TIME and enhances antitumor immunity.

### Anlotinib potentiates IR-stimulated STING activation to facilitate CD8^+^ T cell recruitment

**Fig. 4:**
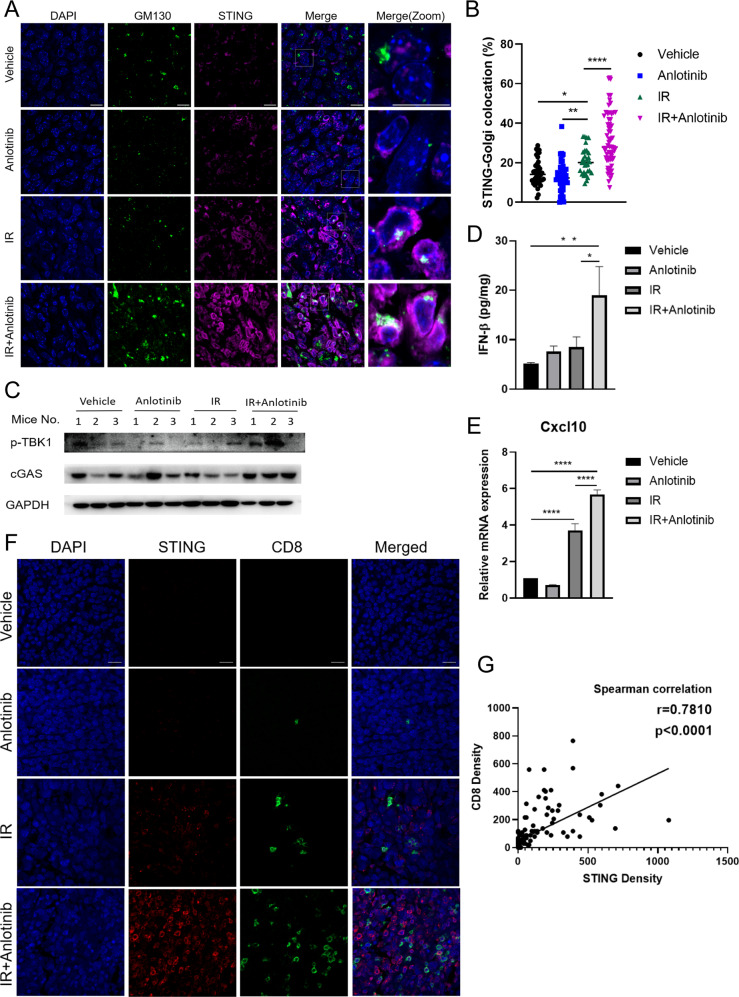
Anlotinib enhances IR-stimulated STING activation to increase CD8^+^ T cell recruitment. **A** Representative images of STING (magenta) localization in cis-Golgi (GM130, green; 63× magnification). “Merge (zoom)” depicts an enlargement of the square above. Scale bar 50 μm. **B** Quantification of the ratio of STING signal localization to the cis-Golgi (GM130) compartment over total STING signal in images as in (**A**), for at least five fields per mouse (*n* = 8–10 mice per group). Each dot represents one field. One-way ANOVA with Tukey’s multiple comparison. **C** Anlotinib combined with IR increased the expression of cGAS and p-TBK1 in subcutaneous tumors from LLC-OVA tumor-bearing mice. **D** The concentration of intratumoral IFN-β was measured using Elisa. One-way ANOVA with Tukey’s multiple comparison. **E** The mRNA expression of *Cxcl10* from tumor lysis was measured via qRT-PCR. Data represent the mean ± standard deviation for 6 mice per group. One-way ANOVA with Tukey’s multiple comparison. **F** Representative multiplex immunofluorescence images of STING (red), CD8^+^ T cells (CD8, green), and nuclear staining (DAPI, blue; ×40 magnification). Scale bar, 20 μm. **G** Correlation of the density of STING^+^ and CD8^+^ cells was assessed using nonparametric Spearman correlation. Every symbol represents one image. *p < 0.05, **p < 0.01, ***p < 0.001, ****p < 0.0001.

We further explored the mechanism of anlotinib affecting CD8^+^ T cell infiltration in vivo. The above data demonstrated that anlotinib enhanced the activation of STING signaling in vitro. STING is activated at the Golgi and the translocation from endoplasmic reticulum to Golgi is essential for the mobilization of STING-dependent type I interferon response [23]. Compared to IR alone, more STING located to GM130, a cis-Golgi marker, following the combination treatment (Fig. 4A, B). Consistently, the expression levels of cGAS and phosphorylated TBK1 were higher in combination treatment in comparison with IR alone (Fig. 4C), indicating that cGAS/STING pathway was further activated following the combination treatment. Moreover, anlotinib combined with IR significantly increased the expression of interferon-beta (IFN-β) triggered by cGAS/STING signaling (Fig. 4D), thus facilitating the activation of CD8^+^ T cells [24]. We also examined mRNA expression of *Ccl5* and *Cxcl10*, two major target genes downstream of STING activation that recruit cytotoxic T lymphocytes to the tumor [22]. The combination treatment remarkably increased the expression levels of *Cxcl10*, but not *Ccl5*, compared to IR alone (Fig. 4E, Supplementary Fig. 5A). In consideration of STING resulting in the activation of both NF-κB- and IRF3-inducible genes, we measured a number of immune genes including NF-κB-dependent genes and IRF3-responsive genes. The combination treatment increased the expression of *Il-8* and *Il-1b*, but it did not increase the expression of other genes mainly activated by NF-κB in comparison with IR alone (Supplementary Fig. 5B). Meanwhile, a significant increase of *Isg15* and *Mx1* gene expression activated by IRF3 was observed in the combination treatment (Supplementary Fig. 5C, D). Together, these data indicated that anlotinib potentiated IR-stimulated type I interferon response mainly through the activation of cGAS-STING-IRF3 signaling cascade. Further, we conducted mIHC to explore the correlation between STING pathway and immune response. The staining results showed a significantly positive correlation between the density of STING and the infiltration of CD8^+^ T cells (Fig. 4F, G). Thus, these data demonstrate that anlotinib combined with IR increases CD8^+^ T cell recruitment mainly through the activation of cGAS-STING-IRF3 signaling cascade.

### Anlotinib enhances the antitumor immunity of radioimmunotherapy

**Fig. 5:**
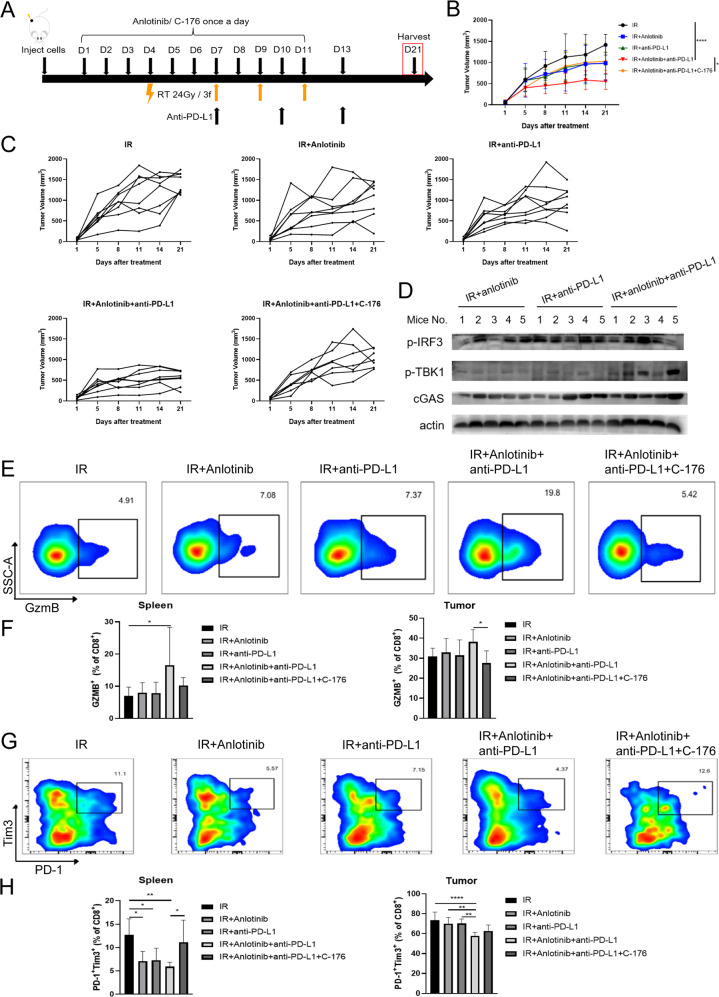
Anlotinib potentiates the antitumor immunity of radioimmunotherapy. **A** Experimental schedule of varied treatment regimens for LLC-OVA tumor-bearing mice. **B** Response of the LLC-OVA subcutaneous tumors to the indicated treatments. Two-way ANOVA with Tukey’s multiple comparison. **C** Individual growth curves for the subcutaneous tumor in each group (IR, *n* = 8; IR + Anlotinib, *n* = 8; IR + anti-PD-L1, *n* = 8; IR + Anlotinib+anti-PD-L1, *n* = 8; IR + Anlotinib+anti-PD-L1 + C-176, *n* = 7). **D** Several key proteins of cGAS/STING signaling were detected in subcutaneous tumors from LLC-OVA tumor-bearing mice treated with IR plus anlotinib, radioimmunotherapy or triple therapy group. **E** Representative flow cytometry staining of CD8^+^ GzmB^+^ T cells in spleen on day 21 after treatment initiation (*n* = 6–8 mice/group). **F** Percentage of CD8^+^ GzmB^+^ T cells in spleen and tumor on day 21. One-way ANOVA with Tukey’s multiple comparison. **G** Representative flow cytometry staining of Tim3 and PD-1 co-expression on CD8^+^ T cells in spleen on day 21. **H** Percentage of CD8^+^ Tim3^+^ PD-1^+^ T cells in spleen and tumor on day 21. One-way ANOVA with Tukey’s multiple comparison. Data represents the mean ± standard deviation, *p < 0.05, **p < 0.01, ***p < 0.001, ****p < 0.0001.

Reportedly, IR combined with ICB can completely cure advanced NSCLC; however, 80% of patients fail to receive a durable benefit, suggesting the need for additional strategies to maximize the efficacy [25]. Whether anlotinib could improve the TIME and enhance the efficacy of radioimmunotherapy through activating STING pathway is yet to be elucidated. Thus, we performed several treatment regimens on LLC-OVA tumor-bearing mice (Fig. 5A). RT combined with anti-PD-L1 and anlotinib (triple therapy) group significantly inhibited tumor growth compared to the radioimmunotherapy and IR plus anlotinib groups, whereas C-176 attenuated the antitumor efficacy of triple therapy (Fig. 5B, C). Moreover, triple therapy increased the expression levels of cGAS and p-TBK1 compared to the radioimmunotherapy and IR plus anlotinib groups (Fig. 5D). These results suggest that anlotinib exerts a synergistic antitumor effect with radioimmunotherapy by activating cGAS/STING pathway.

Next, we explored the effects of triple therapy on TIME and found that triple therapy increased the levels of GzmB in spleen and PB (Fig. 5E, F, Supplementary Fig. 6), whereas the addition of C-176 to triple therapy decreased its content in tumor (Fig. 5F). Moreover, triple therapy reduced the percentage of CD8^+^PD-1^+^Tim3^+^ exhaustion T cells compared to IR, while C-176 reversed the trend in spleen (Fig. 5G, H). In tumor, the percentage of immunosuppressive T cells was lower in triple therapy than that in other groups (Fig. 5H). Together, these results indicate that anlotinib improves the TIME of radioimmunotherapy by enhancing the cytotoxicity of CD8^+^ T cells and reducing the expression of exhausted markers.

### Triple therapy enhances the proliferation and immune memory activation of T cells

**Fig. 6:**
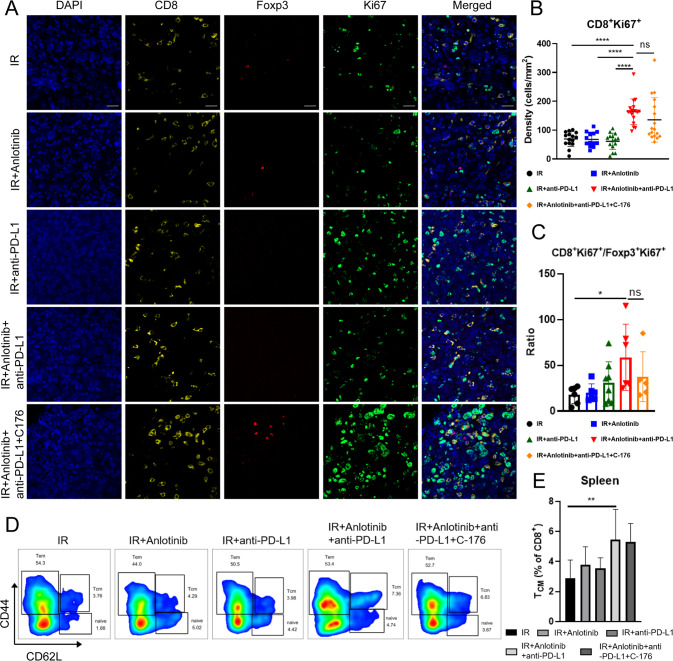
Triple therapy enhances immune memory activation. **A** Representative multiplex immunofluorescence images of CD8^+^ T cells (yellow), Foxp3 (red), Ki67 (green) and nuclear staining (DAPI, blue; 40× magnification). Scale bar, 20 μm. **B** Triple therapy increased the proliferation of tumor-infiltration CD8^+^ T cells. One-way ANOVA with Tukey’s multiple comparison. **C** Triple therapy increased the ratio of CD8^+^ Ki67^+^/Foxp3^+^ Ki67^+^. One-way ANOVA with Tukey’s multiple comparison. **D** Representative flow cytometry staining depicting CD62L and CD44 expression in CD8^+^ T cells in spleen on day 21. **E** Percentage of T_CM_ (CD62L^+^ CD44^+^) of CD8^+^ T cells in spleen on day 21. One-way ANOVA with Tukey’s multiple comparison. Data represents the mean ± standard deviation, *p < 0.05, **p < 0.01, ***p < 0.001, ****p < 0.0001.

Next, we examined the effects of different regimens on T cell proliferation and memory and found that triple therapy maintains a high level of CD8^+^ T cell proliferation (CD8^+^Ki67^+^) (Fig. 6A, B). Moreover, triple therapy increased CD8^+^Ki67^+^/Foxp3^+^Ki67^+^ ratio compared to IR alone (Fig. 6C), indicating that triple therapy fostered immunosupportive TIME. Compared to triple therapy, the addition of C-176 decreased the level of CD8^+^Ki67^+^ and CD8^+^Ki67^+^/Foxp3^+^Ki67^+^, but the difference did not reach significant level. Regarding immune memory, triple therapy boosted the percentage of CD8^+^ central memory T (T_CM_) cells compared to the IR group in spleen (Fig. 6D, E). In summary, triple therapy enhances the proliferation and memory of CD8^+^ T cells that contributes to a durable antitumor response.

## Discussion

In the present study, we found that anlotinib enhanced radiosensitivity in vitro and in vivo. Also, the addition of anlotinib to RT increased the infiltration and activation of IR-stimulated CD8^+^ T cells via activating cGAS/STING signaling. Next, we assessed the synergistic effects of combining anlotinib with RT and anti-PD-L1 and observed that triple therapy significantly attenuated CD8^+^ T cell exhaustion, promoted the cytotoxicity and proliferation of CD8^+^ T cells, and boosted immune memory activation, while the addition of STING inhibitor weakened the trend.

Reportedly, poorly immunogenic tumors have little response to immunotherapy. LLC is a highly aggressive and poorly immunogenic tumor model that is barely responsive to antitumor immunotherapy [26]. LLC-OVA, more immunogenic than LLC, remains resistant to immunotherapy [27]. In the current study, only tumor growth was delayed and exhausted T cells decreased in the radioimmunotherapy group compared to IR alone, whereas CD8^+^ T cell proliferation and immune memory activation were not increased. Moreover, the decrease in Foxp3 expression was not observed in the radioimmunotherapy group compared to IR alone (Supplementary Fig. 7). These findings indicated that radioimmunotherapy could not sufficiently activate antitumor immunity. Conversely, triple therapy significantly reduced CD8^+^ T cell exhaustion and tumor-infiltrating Tregs, and potentiated proliferation, activation, and immune memory of CD8^+^ T cells. Taken together, triple therapy maximally improved TIME compared to other regimens.

The optimal doses of antiangiogenic agents contribute to improving TIME [28]. A previous study shows that the addition of anlotinib (3 mg/kg) boosts the antitumor efficacy of radioimmunotherapy [29]. However, it does not explore the effect of different doses of anlotinib on TIME. In the current study, we found that anlotinib (3 mg/kg) combined with radiation did not increase the percentage of CD8^+^ T cells compared to IR alone (Supplementary Fig. 8). It is reported that anlotinib (1.5 mg/kg) could break through the immunosuppressive barrier to potentiate TIME [30]. Consistently, we found that low-dose (1.5 mg/kg) anlotinib combined with IR could normalize blood vessels (Supplementary Fig. 9). Hence, low-dose (1.5 mg/kg) anlotinib was selected for subsequent experiments. However, the optimal dose for anlotinib is yet controversial and requires further study.

Accumulating evidence suggests that the infiltration and activation of CD8^+^ T cells are closely associated with the prognosis in many types of cancer [31–33]. Furthermore, CD8^+^ cytotoxic T lymphocyte-mediated antitumor immunity is the cornerstone of immune regression of cancer and the determinant of ICB effectiveness [34]. Radiation promotes the infiltration of CD8^+^ T cells into tumors by inducing DSBs to activate the cGAS/STING pathway and potentiates the antitumor immune response by releasing immunogenic tumor-associated antigens and chemokines [8, 35, 36]. In the current study, the addition of anlotinib to IR increased the production of dsDNA, enhanced the expression of crucial proteins of STING pathway, elevated the levels of *Cxcl10*, and ultimately promoted CD8^+^ T cell infiltration. Consistent with our results, some studies suggest that STING is critical for antitumor immune response [37–39]. In addition, STING can also be activated directly by DNA damage response factors ATM and PARP-1 in a cGAS-independent manner, resulting in NF-κB activation, rather than IRF3 [40]. In our work, the non-canonical STING activation appears not to be triggered. Nevertheless, the influence of STING activation on TIME remains controversial. IR-activated cGAS/STING upregulates the expression of PD-L1 in tumor cells that contributes to RT resistance and immune escape [41]. However, elevating tumor PD-L1 expression is a predictive and prognostic factor of immunotherapy response, and the addition of anti-PD-L1 augments the effects of immune-mediated tumor regression [41–43]. On the other hand, published reports document that activation of cGAS/STING also stimulates NF-κB-inducible pro-inflammatory gene expression, which is associated with chronic inflammation, tissue destruction, and tumorigenesis [44, 45]. The activation of STING pathway might discrepantly influence various cancer types. In the present study, we found that triple therapy further activated cGAS/STING signaling, increased CD8^+^ T cell proliferation and enhanced the immune memory activation that facilitated tumor regression and durable control. A previous report demonstrates that STING downregulation is generally observed in human lung cancer subtypes, including small-cell lung cancer, large-cell neuroendocrine lung cancer, LUAD and LUSC, in comparison to normal lung tissues [46]. To further confirm the effect of STING expression on patient survival, we analyzed TCGA dataset of lung cancer. We observed that high expression of STING prolonged the overall survival (OS) in LUAD, while no difference was observed in LUSC (Supplementary Fig. 10). Importantly, the expression of STING was positively correlated with ESTIMATE Score (Supplementary Fig. 11), indicating that STING might promote the infiltration of the immune-stromal component in TIME; however, additional preclinical and prospective studies are required in this field.

The mechanism of tumor regression with respect to RT is complicated. In the current study, we found that cGAS/STING affected the infiltration, activation, and exhaustion of CD8^+^ T cells, but the addition of STING inhibitor had not a significant effect on CD8^+^ T cell proliferation, CD8^+^Ki67^+^/Foxp3^+^Ki67^+^ ratio, Foxp3 expression and immune memory activation compared to triple therapy, indicating that DDR-induced cGAS/STING was not the only regulator. There may be other mechanisms involving in regulating RT-induced TIME, such as endoplasmic reticulum stress, metabolic reprogramming [47, 48], that should be further explored in NSCLC.

Different irradiation fractionations have varied effects on TIME. High-dose radiation increases antigen release and presentation mainly by inducing immunogenic cell death, while low-dose irradiation increases the expression of inducible nitric oxide synthetase on macrophages, which contributes to vascular normalization and T cell recruitment [49]. Moreover, low-dose RT synergistically with combinatorial immunotherapy initiates innate and adaptive immunity to reprogram TIME of immune-desert tumors [50]. Given that the synergistic effects on tumor vascular normalization and immune enhancement, low-dose RT combined with antiangiogenic drugs and immunotherapy would be a promising treatment strategy for “cold” tumors.

RT triggers the innate immune system to activate tumor-specific T cells and increases the diversity of the T-cell receptor repertoire by inducing immunogenic cell death that promotes neoantigen release, which is considered an in situ tumor vaccine [51, 52]. In combination with immunotherapy, local RT generates not only an in situ individualized tumor vaccine, but also abscopal effects [52]. Clinical trials have been designed to irradiate one site to achieve abscopal regression of non-irradiated lesions. In addition, low-dose RT delivered to metastases significantly augments the abscopal effect of high-dose RT combined with ICB [53]. Emerging “multisite” IR irradiates as many sites as possible to achieve total metastatic regression and synergistically boost the efficacy of ICB in improving the OS of metastatic patients [54–56].

In the current study, the addition of anlotinib and anti-PD-L1 boosts the effect of RT as an in situ tumor vaccine, while it is unclear whether and how the treatment affects the abscopal response. The major limitation of this study is that we do not explore the effect of different regimens on the abscopal response. Primary and abscopal tumors exhibit heterogeneities in TIME and may exhibit different responses to the same treatment. In the future study, we will investigate the effect of irradiation on TIME components and abscopal response.

In summary, the current findings indicate the role of anlotinib in modulating antitumor immunity of RT and radioimmunotherapy, which activates cGAS/STING signaling, promotes the infiltration and activation of CD8^+^ T cells, potentiates the proliferation and immune memory of CD8^+^ T cells, and eventually makes tumor more responsive to radiation and radioimmunotherapy. The addition of anlotinib to radioimmunotherapy might be a promising strategy to amplify the benefit for NSCLC patients.

## Materials and methods

### Cell culture and reagents

Human NSCLC cell lines (A549 and H460) were obtained from the Cell Resource Center, Peking Union Medical College (Beijing, China), H1299 was purchased from the ATCC, and other human NSCLC cell lines (H292, H2228, HCC827, H1703, and Calu-1) were purchased from Shanghai Cell Bank of Chinese Academy of Sciences (Shanghai, China). The murine Lewis lung carcinoma (LLC) cell line was obtained from the ATCC. The LLC-OVA cell line expressing a single polypeptide encoding H-2Kbb2-M, and the OVA SIINFEKL peptide was kindly gifted by Amer A. Beg (Moffitt Cancer Center, Tampa, FL). H460, H1299, H292, H2228, HCC827 and H1703 cell lines were cultured in RPMI 1640 medium containing 10% fetal bovine serum (FBS) and 1% penicillin-streptomycin. Calu-1 cells were cultivated in McCoy’s 5A medium supplemented with 10% FBS and 1% penicillin-streptomycin. A549 and LLC-OVA cells were cultured in DMEM medium supplemented with 10% FBS and 1% penicillin-streptomycin. All the cell lines were cultured in a 37 °C humidified incubator with 5% CO_2_.

Anlotinib was kindly provided by Chia Tai Tianqing Pharmaceutical Group Co., Ltd (Nanjing, Jiangsu, China) and was prepared as a 10 mmol/L stock solution in dimethyl sulfoxide (DMSO) for in vitro experiments or in normal saline for in vivo studies.

### In vivo mouse models

All studies were supervised and approved by the Ethics Committee of Tianjin Medical University Cancer Hospital and Institute. All mice were purchased from SiPeiFu (Beijing, China) and maintained under specific pathogen-free conditions.

### Nude Mouse Model

Tumor xenografts were established by subcutaneous injection of 5 × 10^6^ H460 cells into the right proximal hind legs of the female BALB/c nude mice (6 weeks old). When tumors grew to a volume of approximately 200 mm^3^, mice were randomly divided into the following four groups: control (*n* = 4), anlotinib (*n* = 4), IR (*n* = 4), and IR plus anlotinib (*n* = 3). Specifically, mice were administered normal saline or 0.75 mg/kg anlotinib via gavage daily for 14 days. A 10 Gy radiation dose (2 Gy once daily in 5 fractions) was delivered locally to the tumor after 3 days of gavage using a photon beam linear accelerator (6MV-X-ray) (Varian, USA). Tumor dimensions were measured with vernier caliper every three days, and tumor volume was calculated using V = length×width^2^/2. All mice were euthanized at 22 days.

### Syngeneic Mouse Model

Murine LLC-OVA cells (2 × 10^6^) were subcutaneously injected into the right proximal hind legs of the female C57BL/6 mice (6 weeks old). The mice were divided into different treatment groups when tumor volume was approximately 100 mm^3^. Tumor volume was calculated using *V* = length×width^2^/2. For radiotherapy experiment, 3 fractions of 8 Gy were delivered locally to the tumor every other day. For anlotinib treatment, mice were given anlotinib (1.5 mg/kg) by daily gavage from one week before IR until the complete of radiation. For ICB treatment, the anti-PD-L1 (200 μg/mouse, B7-H1, clone 10 F.9G2, Bio X Cell) or isotype control IgG was intraperitoneally injected every three days from the start of radiation. For C-176 (STING inhibitor) administration, mice were intraperitoneally injected with C-176 (S6575, Selleck) daily with a dose of 5 mg/kg starting one week before irradiation till the end of RT. For ethical considerations, mice were sacrificed when tumor volume reached approximately 2 000 mm^3^ or showed signs of morbidity.

### Cell viability assay

H460 and A549 cells were seeded into 96-well plates and incubated with various concentrations of anlotinib, and then cell viability was evaluated using the Cell Counting Kit-8 (CCK-8, MedChemExpress, USA). The absorbance was measured at 450 nm at the indicated time point. Experiments were performed in triplicates.

### Colony formation assay

H460 and A549 cells were pretreated with anlotinib or DMSO for 48 h and then were seeded at 100 to 800 cells per well in sex-well plates for colony formation. Cells were irradiated with varying doses of 0, 2, 4, and 6 Gy. After 24 h of treatment with IR, the supernatant was replaced with fresh medium to maintain colony formation. Ten to 14 days after irradiation, cells were fixed with formaldehyde for 15 min, and then stained with 0.5% crystal violet for 10 min at room temperature. Colonies containing 50 or more cells were counted. Plating efficiency was defined as the mean number of colonies counted/number of cells seeded. The surviving fraction was calculated as follows: mean number of colonies counted/(number of cells plated *plating efficiency). The cell survival curves were fitted with the linear-quadratic model using GraphPad Prism 8.0 in formula *Y* = exp(−(a*x + b*(*x*^2^))).

### Western blot analysis

Proteins were extracted from different cells by using cell lysis buffer with protease and phosphatase inhibitors (Solarbio, Beijing, China) according to the manufacturer’s instructions, and protein concentration was quantified using a BCA Protein Assay Kit (Thermo Fisher Scientific, USA). Proteins were separated by sodium dodecyl sulfate-polyacrylamide gel electrophoresis and then transferred to polyvinylidene fluoride membrane (Millipore, USA). Membranes were incubated with antibodies, and the ECL detection reagent (Thermo Fisher Scientific) was used to detect signal. Detailed antibodies information was listed in Supplementary Table 1.

### Immunofluorescence assay

Cells were spread on coverslips in 24-well plates at 4 × 10^4^ cells per well, pretreated with DMSO or anlotinib for 48 h, and then irradiated with 4 Gy X-ray. At the indicated time point after radiation, the cells were fixed with 4% paraformaldehyde, permeabilized with 0.2% Triton X-100, and then incubated with primary antibodies overnight at 4 °C. Next, cells were incubated with fluorescent secondary antibody for 1 h at room temperature and then stained with 4′,6-diamidino-2-phenylindole (DAPI; Thermo Fisher Scientific). Finally, stained sections were observed and images were acquired using a Zeiss Imaginer-Z2 (Jena, Germany). Detailed antibodies information was listed in Supplementary Table 1.

### PicoGreen staining

Cells were pretreated with DMSO or anlotinib for 48 h, and then irradiated with 4 Gy X-ray. After 48 h, cells were incubated in medium containing PicoGreen (ThermoFisher, P11496) 3 μl/ml for 1 h in a 37 °C humidified incubator. The cells were washed and fixed, and then counterstained with DAPI for confocal microscopy.

### Flow cytometry analysis

Lymphocytes were extracted from peripheral blood (PB), tumors and spleens as previously described [9, 25]. Briefly, PB, tumor tissues and spleens from mice were prepared to single-cell suspensions and stained with antibodies (detail was listed in Supplementary Table 1). Cell surface staining was conducted via incubation for 30 min at room temperature. For intracellular staining, the cells were fixed and then permeabilized with an intracellular staining/permeabilization solution (BioLegend, San Diego, CA, USA). Then, the cells were stained with intracellular antibodies for 30 min at room temperature. Data were acquired using an LSRFortessa flow cytometer (BD Biosciences), and analyzed with the FlowJo 10 software.

### Quantitative real-time PCR (qRT-PCR)

Total RNA was extracted from tumor tissues using Cell/Tissue Total RNA Kit (YEASEN, China) in accordance with the manufacturer’s protocol. Then cDNA was synthesized using PrimeScript™ RT reagent Kit (Perfect Real Time) (Takara, RR037A). With TB Green® Premix Ex Taq™ II (Tli RNaseH Plus) (Takara, RR820A), qRT-PCR was performed using the Real-Time PCR Detection system (Roche). Relative mRNA levels were calculated using the –ΔΔCt method (*Gapdh* or *β-Actin* gene as control) and were expressed as 2^−ΔΔCt^. A list of primers was indicated in Supplementary Table 2.

### Enzyme linked immunosorbent assay (Elisa)

Tumor tissues were cut into small pieces, and soaked in lysis buffer containing protease inhibitors, then processed using the tissue homogenizer. The supernatants from samples were harvested for the subsequent experiments. Intratumoral IFN-β and IFN-γ concentrations were measured using Elisa kit (MultiSciences, 70-EK2236; Thermo Fisher, 88-7314-88) following the manufacturer’s guidelines. Relative levels of IFN-β and IFN-γ were calculated by normalizing to protein concentration of each sample.

### Immunohistochemistry (IHC) assay and Multiplexed IHC (mIHC) staining

Deparaffinized and rehydrated tumor sections were placed in ethylenediaminetetraacetic acid (EDTA) (pH 8.0), and antigen retrieval was performed using microwave treatment (MWT) for 15 min. After blocking with 3% hydrogen peroxide and normal goat serum, the slides were incubated with Granzyme B (GzmB) or CD31 overnight at 4 °C. Next, sections were stained with the secondary antibody followed by DAB kit (#ZL1-9019, ZSGB-BIO) to amplify and detect signals, and then counterstained with hematoxylin. Images were acquired from 3–10 non-overlapping areas per section with Zeiss Imaginer at 200× magnification.

For mIHC staining, Opal 7-color fluorescent IHC kit (PerkinElmer, NEL81001KT) was used. The stanning panels were as follows: GM130/TSA 520, STING/TSA 690 and DAPI; CD8/TSA 520, STING/TSA 690 and DAPI; CD8/TSA 690, Foxp3/TSA 620, Ki67/TSA 520 and DAPI. In the first staining cycle, deparaffinized and rehydrated sections were immersed in EDTA (pH 8.0) and treated with MWT 15 min for antigen retrieval. Then, sections were incubated with blocking buffer and primary antibodies, following by secondary-HRP and fluorescent TSA reagent. The primary antibody-TSA complex was removed by MWT and the staining process was repeated for a subsequent target. At last, DAPI was used for cell nuclei staining. Finally, sections were scanned and visualized with a Zeiss LSM800 confocal laser scanning microscope system with the same exposure times and intensity. Details of antibodies were described in Supplementary Table 1.

For positive cells quantitation (GzmB) in IHC, the numbers of GzmB^+^ cells were counted manually, and the results were described as the number of positive cells per field. For mIHC, 5–15 random areas without hemorrhage, necrosis, or detachment were selected and analyzed. The positive cell numbers of CD8, Foxp3 and Ki67 per mm^2^ were quantified using ImageJ software [57, 58]. The results were expressed as the density (positive cells per mm^2^) per section. Quantification analysis of STING/GM130 colocalization was performed using ImageJ Fiji (version 2.1.0). Colocalization calculation method is based on previous literature [59].

### Kaplan–Meier survival analysis

The survival data and mRNA expression of STING (TMEM173) of lung adenocarcinoma (LUAD) and lung squamous cell carcinoma (LUSC) were acquired from The Cancer Genome Atlas (TCGA). X-tile (Yale University, version 3.6.1) was used to determine the optimum cutoff of STING expression. The overall survival curves associated with STING expression status (high versus low) and *p*-value were generated with survminer (0.4.9) package.

### Statistical analyses

A two-tailed, unpaired *t* test was used to compare two groups. Statistical analysis of more than two groups was performed using one-way analysis of variance with Tukey’s multiple comparison test. Two-way analysis of variance was applied for tumor growth analysis. Log-rank test was used for survival analysis. Statistical analyses and plot graphs were performed using GraphPad Prism 8.0 software (GraphPad, San Diego, California, USA). Data are represented as mean ± standard deviation. *P* < 0.05 was considered statistically significant.

## Supplementary information

Supplementary Figure and Table Legends

Supplementary Figure 1

Supplementary Figure 2

Supplementary Figure 3

Supplementary Figure 4

Supplementary Figure 5

Supplementary Figure 6

Supplementary Figure 7

Supplementary Figure 8

Supplementary Figure 9

Supplementary Figure 10

Supplementary Figure 11

Supplementary Table 1

Supplementary Table 2

Original Data File

## Data Availability

All data generated or analyzed during this study are included in this published article and its supplementary information files.

## References

[1] Bray F, Ferlay J, Soerjomataram I, Siegel RL, Torre LA, Jemal A (2018). Global cancer statistics 2018: GLOBOCAN estimates of incidence and mortality worldwide for 36 cancers in 185 countries. CA Cancer J Clin.

[2] Duma N, Santana-Davila R, Molina JR (2019). Non-small cell lung cancer: epidemiology, screening, diagnosis, and treatment. Mayo Clin Proc.

[3] Koning CC, Wouterse SJ, Daams JG, Uitterhoeve LL, van den Heuvel MM, Belderbos JS (2013). Toxicity of concurrent radiochemotherapy for locally advanced non–small-cell lung cancer: a systematic review of the literature. Clin Lung Cancer.

[4] Zhao H, Xie Y-Z, Xing R, Sun M, Chi F, Zeng Y-C (2017). MDMX is a prognostic factor for non-small cell lung cancer and regulates its sensitivity to cisplatin. Cell Oncol (Dordr).

[5] Theelen W, Chen D, Verma V, Hobbs BP, Peulen HMU, Aerts J (2021). Pembrolizumab with or without radiotherapy for metastatic non-small-cell lung cancer: a pooled analysis of two randomised trials. Lancet Respiratory Med.

[6] Theelen W, Peulen HMU, Lalezari F, van der Noort V, de Vries JF, Aerts J (2019). Effect of pembrolizumab after stereotactic body radiotherapy vs pembrolizumab alone on tumor response in patients with advanced non-small cell lung cancer: results of the PEMBRO-RT phase 2 Randomized Clinical Trial. JAMA Oncol.

[7] Schoenfeld JD, Giobbie-Hurder A, Ranasinghe S, Kao KZ, Lako A, Tsuji J (2022). Durvalumab plus tremelimumab alone or in combination with low-dose or hypofractionated radiotherapy in metastatic non-small-cell lung cancer refractory to previous PD(L)-1 therapy: an open-label, multicentre, randomised, phase 2 trial. Lancet Oncol.

[8] Huang Y, Sheng H, Xiao Y, Hu W, Zhang Z, Chen Y (2021). Wnt/beta-catenin inhibitor ICG-001 enhances the antitumor efficacy of radiotherapy by increasing radiation-induced DNA damage and improving tumor immune microenvironment in hepatocellular carcinoma. Radiother Oncol.

[9] Sheng H, Huang Y, Xiao Y, Zhu Z, Shen M, Zhou P, et al. ATR inhibitor AZD6738 enhances the antitumor activity of radiotherapy and immune checkpoint inhibitors by potentiating the tumor immune microenvironment in hepatocellular carcinoma. J Immunother Cancer. 2020;8:e000340. 10.1136/jitc-2019-000340 PMC7254123 32461345

[10] Deng L, Liang H, Xu M, Yang X, Burnette B, Arina A (2014). STING-dependent cytosolic DNA sensing promotes radiation-induced type I interferon-dependent antitumor immunity in immunogenic tumors. Immunity.

[11] Sato H, Niimi A, Yasuhara T, Permata TBM, Hagiwara Y, Isono M (2017). DNA double-strand break repair pathway regulates PD-L1 expression in cancer cells. Nat Commun.

[12] Dovedi SJ, Adlard AL, Lipowska-Bhalla G, McKenna C, Jones S, Cheadle EJ (2014). Acquired resistance to fractionated radiotherapy can be overcome by concurrent PD-L1 blockade. Cancer Res.

[13] Sun Y, Niu W, Du F, Du C, Li S, Wang J (2016). Safety, pharmacokinetics, and antitumor properties of anlotinib, an oral multi-target tyrosine kinase inhibitor, in patients with advanced refractory solid tumors. J Hematol Oncol.

[14] Lin B, Song X, Yang D, Bai D, Yao Y, Lu N (2018). Anlotinib inhibits angiogenesis via suppressing the activation of VEGFR2, PDGFRβ and FGFR1. Gene.

[15] Liao J, Jin H, Li S, Xu L, Peng Z, Wei G (2019). Apatinib potentiates irradiation effect via suppressing PI3K/AKT signaling pathway in hepatocellular carcinoma. J Exp Clin Cancer Res.

[16] Gao H, Xue J, Zhou L, Lan J, He J, Na F (2015). Bevacizumab radiosensitizes non-small cell lung cancer xenografts by inhibiting DNA double-strand break repair in endothelial cells. Cancer Lett.

[17] Khan KA, Kerbel RS (2018). Improving immunotherapy outcomes with anti-angiogenic treatments and vice versa. Nat Rev Clin Oncol.

[18] Fukumura D, Kloepper J, Amoozgar Z, Duda DG, Jain RK (2018). Enhancing cancer immunotherapy using antiangiogenics: opportunities and challenges. Nat Rev Clin Oncol.

[19] Dillon MT, Good JS, Harrington KJ (2014). Selective targeting of the G2/M cell cycle checkpoint to improve the therapeutic index of radiotherapy. Clin Oncol (R Coll Radiologists (Gt Br)).

[20] Panier S, Boulton SJ (2014). Double-strand break repair: 53BP1 comes into focus. Nat Rev Mol Cell Biol.

[21] Klinakis A, Karagiannis D, Rampias T (2020). Targeting DNA repair in cancer: current state and novel approaches. Cell Mol Life Sci: CMLS.

[22] Shen J, Zhao W, Ju Z, Wang L, Peng Y, Labrie M (2019). PARPi triggers the STING-dependent immune response and enhances the therapeutic efficacy of immune checkpoint blockade independent of BRCAness. Cancer Res.

[23] Mukai K, Konno H, Akiba T, Uemura T, Waguri S, Kobayashi T (2016). Activation of STING requires palmitoylation at the Golgi. Nat Commun.

[24] Yu R, Zhu B, Chen D (2022). Type I interferon-mediated tumor immunity and its role in immunotherapy. Cell Mol Life Sci: CMLS.

[25] Reijmen E, De Mey S, De Mey W, Gevaert T, De Ridder K, Locy H (2021). Fractionated radiation severely reduces the number of CD8+ T cells and mature antigen presenting cells within lung tumors. Int J Radiat Oncol Biol Phys.

[26] Lechner MG, Karimi SS, Barry-Holson K, Angell TE, Murphy KA, Church CH (2013). Immunogenicity of murine solid tumor models as a defining feature of in vivo behavior and response to immunotherapy. J Immunother.

[27] Chamoto K, Takeshima T, Wakita D, Ohkuri T, Ashino S, Omatsu T (2009). Combination immunotherapy with radiation and CpG-based tumor vaccination for the eradication of radio- and immuno-resistant lung carcinoma cells. Cancer Sci.

[28] Zhao S, Ren S, Jiang T, Zhu B, Li X, Zhao C (2019). Low-dose apatinib optimizes tumor microenvironment and potentiates antitumor effect of PD-1/PD-L1 blockade in lung cancer. Cancer Immunol Res.

[29] Yuan M, Zhai Y, Men Y, Zhao M, Sun X, Ma Z (2022). Anlotinib enhances the antitumor activity of high-dose irradiation combined with anti-PD-L1 by potentiating the tumor immune microenvironment in murine lung cancer. Oxid Med Cell Longev.

[30] Liu S, Qin T, Liu Z, Wang J, Jia Y, Feng Y (2020). anlotinib alters tumor immune microenvironment by downregulating PD-L1 expression on vascular endothelial cells. Cell Death Dis.

[31] Jie X, Chen Y, Zhao Y, Yang X, Xu Y, Wang J, et al. Targeting KDM4C enhances CD8(+) T cell mediated antitumor immunity by activating chemokine CXCL10 transcription in lung cancer. J Immunother Cancer. 2022;10:e003716. 10.1136/jitc-2021-003716 PMC8819819 35121645

[32] Byrne A, Savas P, Sant S, Li R, Virassamy B, Luen SJ (2020). Tissue-resident memory T cells in breast cancer control and immunotherapy responses. Nat Rev Clin Oncol.

[33] Li X, Gruosso T, Zuo D, Omeroglu A, Meterissian S, Guiot MC (2019). Infiltration of CD8(+) T cells into tumor cell clusters in triple-negative breast cancer. Proc Natl Acad Sci USA.

[34] Li JY, Chen YP, Li YQ, Liu N, Ma J (2021). Chemotherapeutic and targeted agents can modulate the tumor microenvironment and increase the efficacy of immune checkpoint blockades. Mol Cancer.

[35] Vanpouille-Box C, Alard A, Aryankalayil MJ, Sarfraz Y, Diamond JM, Schneider RJ (2017). DNA exonuclease Trex1 regulates radiotherapy-induced tumour immunogenicity. Nat Commun.

[36] Marciscano AE, Ghasemzadeh A, Nirschl TR, Theodros D, Kochel CM, Francica BJ (2018). Elective nodal irradiation attenuates the combinatorial efficacy of stereotactic radiation therapy and immunotherapy. Clin Cancer Res.

[37] Harding SM, Benci JL, Irianto J, Discher DE, Minn AJ, Greenberg RA (2017). Mitotic progression following DNA damage enables pattern recognition within micronuclei. Nature.

[38] Wang H, Hu S, Chen X, Shi H, Chen C, Sun L (2017). cGAS is essential for the antitumor effect of immune checkpoint blockade. Proc Natl Acad Sci USA.

[39] Long ZJ, Wang JD, Xu JQ, Lei XX, Liu Q (2022). cGAS/STING cross-talks with cell cycle and potentiates cancer immunotherapy. Mol Ther.

[40] Dunphy G, Flannery SM, Almine JF, Connolly DJ, Paulus C, Jonsson KL (2018). Non-canonical activation of the DNA sensing adaptor STING by ATM and IFI16 mediates NF-kappaB signaling after nuclear DNA damage. Mol Cell.

[41] Du SS, Chen GW, Yang P, Chen YX, Hu Y, Zhao QQ (2022). Radiation therapy promotes hepatocellular carcinoma immune cloaking via PD-L1 upregulation induced by cGAS-STING activation. Int J Radiat Oncol Biol Phys.

[42] Bryant AK, Sankar K, Strohbehn GW, Zhao L, Daniel V, Elliott D (2022). Prognostic and predictive role of PD-L1 expression in stage III non-small cell lung cancer treated with definitive chemoradiation and adjuvant durvalumab. Int J Radiat Oncol Biol Phys.

[43] Herbst RS, Baas P, Kim D-W, Felip E, Pérez-Gracia JL, Han J-Y (2016). Pembrolizumab versus docetaxel for previously treated, PD-L1-positive, advanced non-small-cell lung cancer (KEYNOTE-010): a randomised controlled trial. Lancet.

[44] Ahn J, Xia T, Konno H, Konno K, Ruiz P, Barber GN (2014). Inflammation-driven carcinogenesis is mediated through STING. Nat Commun.

[45] Dou Z, Ghosh K, Vizioli MG, Zhu J, Sen P, Wangensteen KJ (2017). Cytoplasmic chromatin triggers inflammation in senescence and cancer. Nature.

[46] Marinello J, Arleo A, Russo M, Delcuratolo M, Ciccarelli F, Pommier Y (2022). Topoisomerase I poison-triggered immune gene activation is markedly reduced in human small-cell lung cancers by impairment of the cGAS/STING pathway. Br J Cancer.

[47] Yang W, Xiu Z, He Y, Huang W, Li Y, Sun T (2020). Bip inhibition in glioma stem cells promotes radiation-induced immunogenic cell death. Cell Death Dis.

[48] Lai YC, Hsieh CY, Lu KY, Sung CH, Ho HY, Cheng ML, et al. Monitoring early glycolytic flux alterations following radiotherapy in cancer and immune cells: hyperpolarized carbon-13 magnetic resonance imaging study. metabolites. 2021;11:518. 10.3390/metabo11080518 PMC8398834 34436459

[49] Klug F, Prakash H, Huber PE, Seibel T, Bender N, Halama N (2013). Low-dose irradiation programs macrophage differentiation to an iNOS^+^/M1 phenotype that orchestrates effective T cell immunotherapy. Cancer Cell.

[50] Herrera FG, Ronet C, Ochoa de Olza M, Barras D, Crespo I, Andreatta M (2022). Low-dose radiotherapy reverses tumor immune desertification and resistance to immunotherapy. Cancer Discov.

[51] Twyman-Saint Victor C, Rech AJ, Maity A, Rengan R, Pauken KE, Stelekati E (2015). Radiation and dual checkpoint blockade activate non-redundant immune mechanisms in cancer. Nature.

[52] Demaria S, Golden EB, Formenti SC (2015). Role of local radiation therapy in cancer immunotherapy. JAMA Oncol.

[53] Yin L, Xue J, Li R, Zhou L, Deng L, Chen L (2020). Effect of low-dose radiation therapy on abscopal responses to hypofractionated radiation therapy and anti-PD1 in mice and patients with non-small cell lung cancer. Int J Radiat Oncol Biol Phys.

[54] Arina A, Gutiontov SI, Weichselbaum RR (2020). Radiotherapy and immunotherapy for cancer: from “systemic” to “multisite”. Clin Cancer Res.

[55] Luke JJ, Lemons JM, Karrison TG, Pitroda SP, Melotek JM, Zha Y (2018). Safety and clinical activity of pembrolizumab and multisite stereotactic body radiotherapy in patients with advanced solid tumors. J Clin Oncol.

[56] Demaria S, Guha C, Schoenfeld J, Morris Z, Monjazeb A, Sikora A, et al. Radiation dose and fraction in immunotherapy: one-size regimen does not fit all settings, so how does one choose? J Immunother Cancer. 2021;9:e002038. 10.1136/jitc-2020-002038 PMC8031689 33827904

[57] Zhang QF, Li J, Jiang K, Wang R, Ge JL, Yang H (2020). CDK4/6 inhibition promotes immune infiltration in ovarian cancer and synergizes with PD-1 blockade in a B cell-dependent manner. Theranostics.

[58] Ying L, Yan F, Meng Q, Yu L, Yuan X, Gantier MP, et al. PD-L1 expression is a prognostic factor in subgroups of gastric cancer patients stratified according to their levels of CD8 and FOXP3 immune markers. OncoImmunology. 2018;7:e1433520. 10.1080/2162402X.2018.1433520 PMC5980489 29872566

[59] Lepelley A, Martin-Niclos MJ, Le Bihan M, Marsh JA, Uggenti C, Rice GI, et al. Mutations in COPA lead to abnormal trafficking of STING to the Golgi and interferon signaling. J Exp Med. 2020;217:e20200600. 10.1084/jem.20200600 PMC7596811 32725128

