# Supplementary material for: Anlotinib enhances the antitumor immunity of radiotherapy by activating cGAS/STING in non-small cell lung cancer
Source: Cell Death Discov. 2022 Nov 28;8:468. doi: 10.1038/s41420-022-01256-2 (PMC9705441; doi:10.1038/s41420-022-01256-2)
Supplement: Supplementary file 1 — Supplementary Figure and Table Legends [file 41420_2022_1256_MOESM1_ESM.docx]

**Supplementary** **Figure Legends**

**Supplementary Fig. 1** H460 and A59 cells were incubated with different concentrations of anlotinib for 24, 48, and 72 h, and then cell viability was examined by CCK-8.

**Supplementary Fig. 2** Hematoxylin and eosin staining of internal organs (including lung, liver, heart, kidney, and spleen) from euthanized mice were performed.

**Supplementary Fig. 3** Detection of 53BP1 foci formation was performed at 0h and 24h after irradiation (4 Gy). Student unpaired t test. Data represent the mean ± standard deviation, *p < 0.05, ** p < 0.01, *** p < 0.001, **** p < 0.0001.

**Supplementary Fig. 4** The expression of cGAS and STING in different NSCLC cell lines.

**Supplementary Fig. 5** The mRNA expression levels of immune genes activated by IRF3 **(A, C, D)** and NF-κB **(B)** from tumor lysis were measured via qRT-PCR. Data represent the mean ± standard deviation for 5-8 mice per group. One-way ANOVA with Tukey’s multiple comparison. *p < 0.05, ** p < 0.01, *** p < 0.001, **** p < 0.0001, ns = not significant.

**Supplementary Fig. 6** Percentage of CD8^+^ GzmB^+^ T cells in PB on day 21. One-way ANOVA with Tukey’s multiple comparison. Data represent the mean ± standard deviation, *p < 0.05, ** p < 0.01, *** p < 0.001. PB, peripheral blood.

**Supplementary Fig. 7** Quantification of Foxp3 expression. Triple therapy decreased tumor-infiltrating Tregs. Each dot represents one field. One-way ANOVA with Tukey’s multiple comparison. Data represent the mean ± standard deviation, *p < 0.05.

**Supplementary Fig. 8** LLC-OVA tumor-bearing mice were treated with anlotinib (3 mg/kg) or IR (8Gy × 3f) (n= 3 mice/group). Representative images and quantification of CD8^+^ T cells in spleen on day 16 after treatment initiation. One-way ANOVA with Tukey’s multiple comparison. Data represent the mean ± standard deviation, ns = not significant.

**Supplementary Fig. 9** Low-dose anlotinib (1.5 mg/kg) combined with IR decreased the microvessel density. Representative images of CD31 from tumor tissues. Scale bar, 200 μm.

**Supplementary Fig. 10** Overall survival of LUAD (576) and LUSC (552) patients from TCGA stratified by STING expression. Log-rank test. LUAD, lung adenocarcinoma; LUSC, lung squamous cell carcinoma.

**Supplementary Fig. 11** The assessment of immune-stromal component in LUAD of TCGA. Mann-Whitney t test. **** p < 0.0001. LUAD, lung adenocarcinoma.

**Supplementary** **Table Legends**

**Supplemental Table 1** Antibodies for western blot (WB), immunofluorescence (IF), immunohistochemistry (IHC) and flow cytometry (FC).

**Supplementary Table 2** Sequences of forward and reverse primers used for qRT-PCR.
