# Supplementary figures and images for: Anlotinib enhances the antitumor immunity of radiotherapy by activating cGAS/STING in non-small cell lung cancer
Source: Cell Death Discov. 2022 Nov 28;8:468. doi: 10.1038/s41420-022-01256-2 (PMC9705441; doi:10.1038/s41420-022-01256-2)

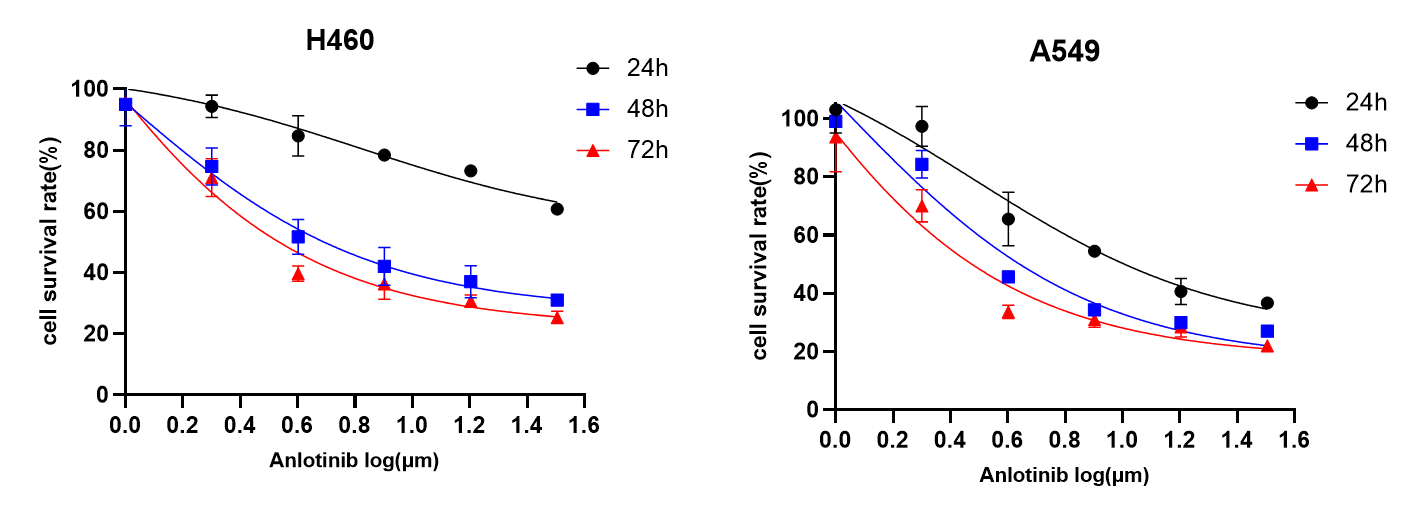

Supplement: Supplementary file 2 — Supplementary Figure 1 [file 41420_2022_1256_MOESM2_ESM.tif]

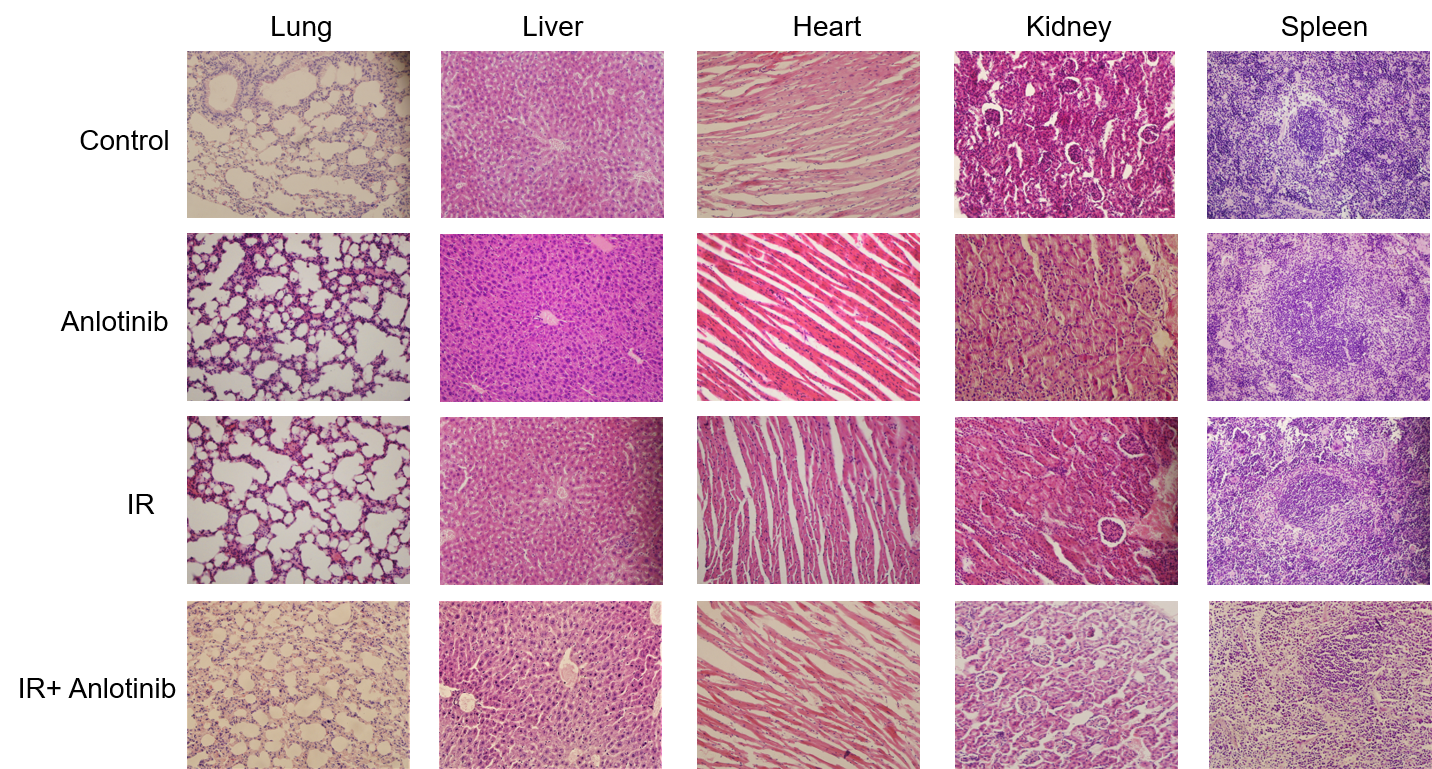

Supplement: Supplementary file 3 — Supplementary Figure 2 [file 41420_2022_1256_MOESM3_ESM.tif]

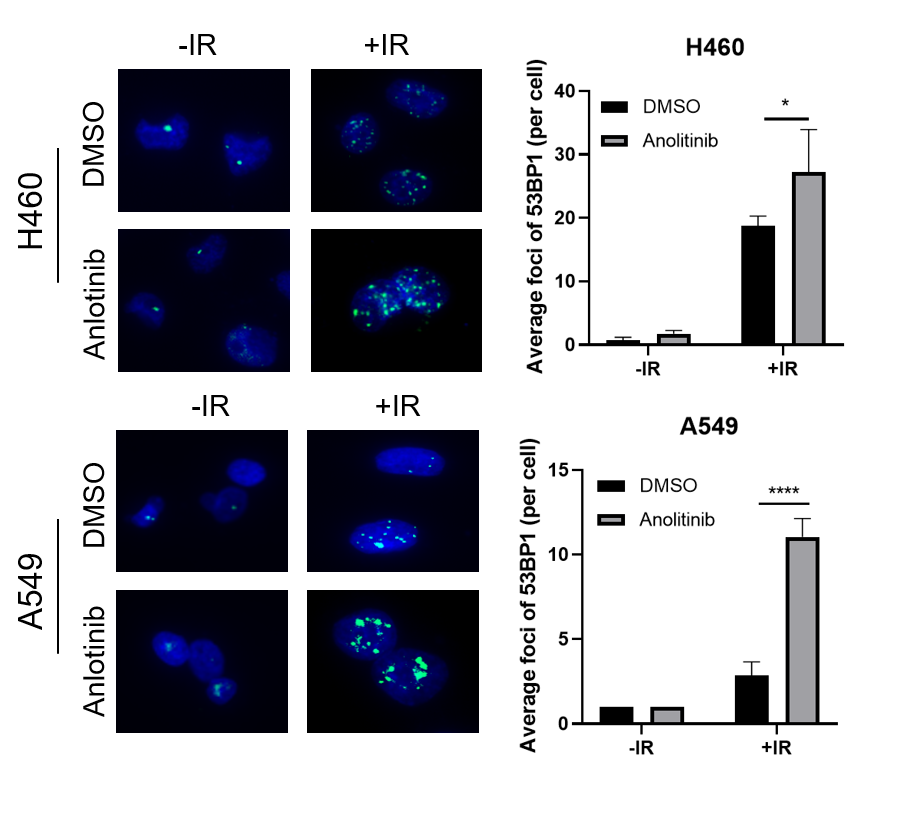

Supplement: Supplementary file 4 — Supplementary Figure 3 [file 41420_2022_1256_MOESM4_ESM.tif]

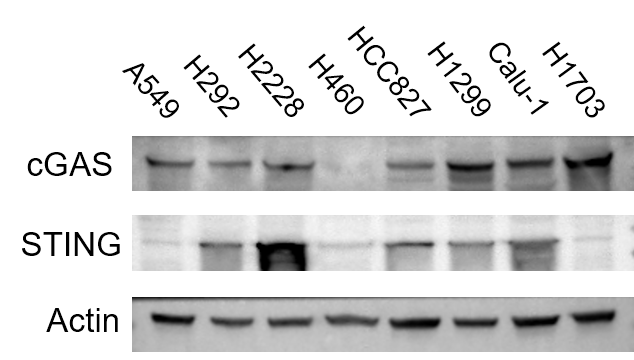

Supplement: Supplementary file 5 — Supplementary Figure 4 [file 41420_2022_1256_MOESM5_ESM.tif]

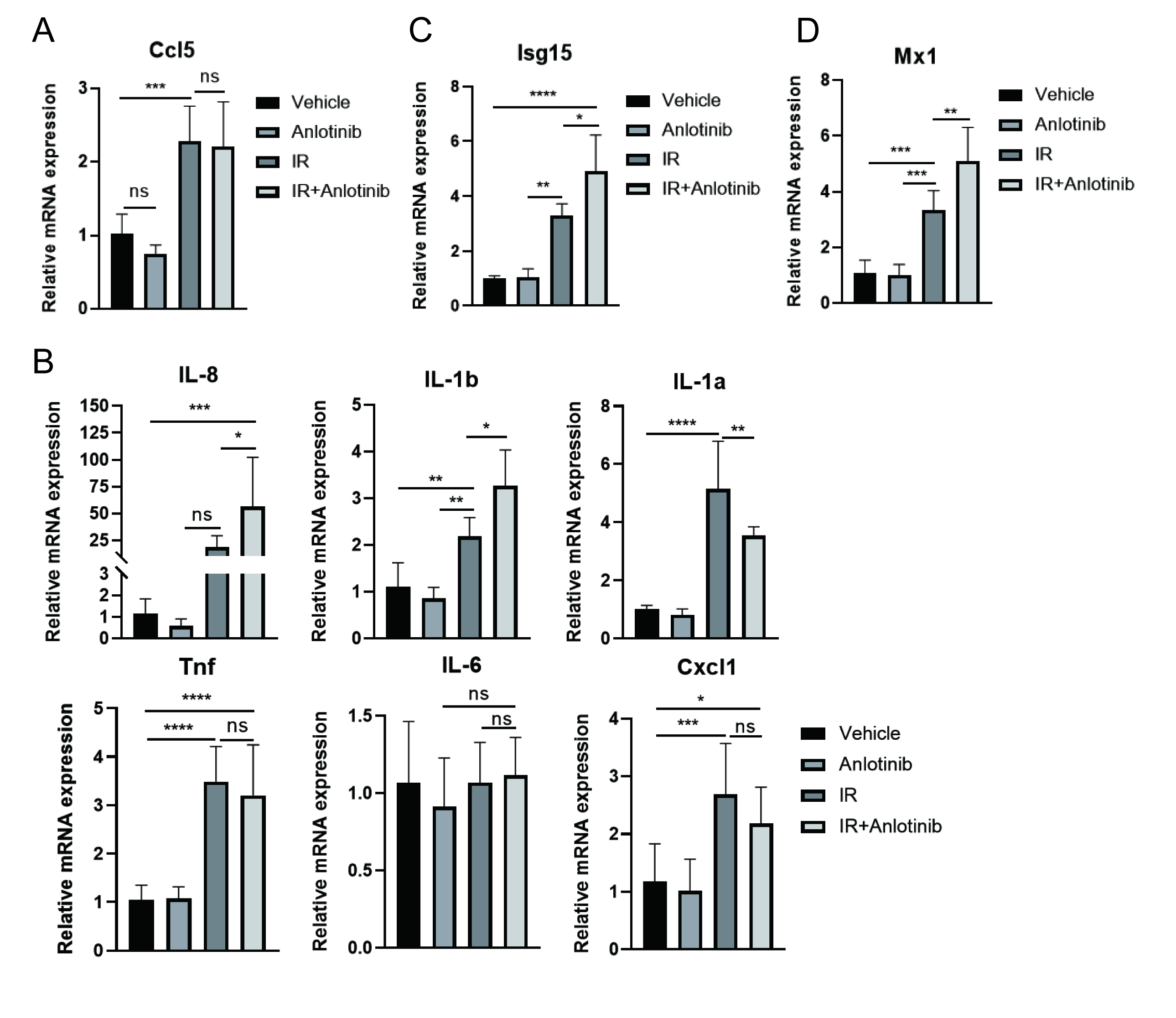

Supplement: Supplementary file 6 — Supplementary Figure 5 [file 41420_2022_1256_MOESM6_ESM.tif]

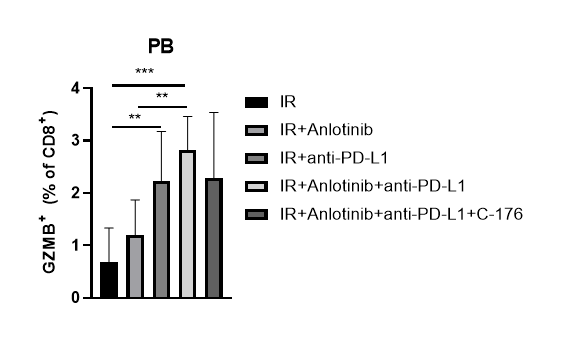

Supplement: Supplementary file 7 — Supplementary Figure 6 [file 41420_2022_1256_MOESM7_ESM.tif]

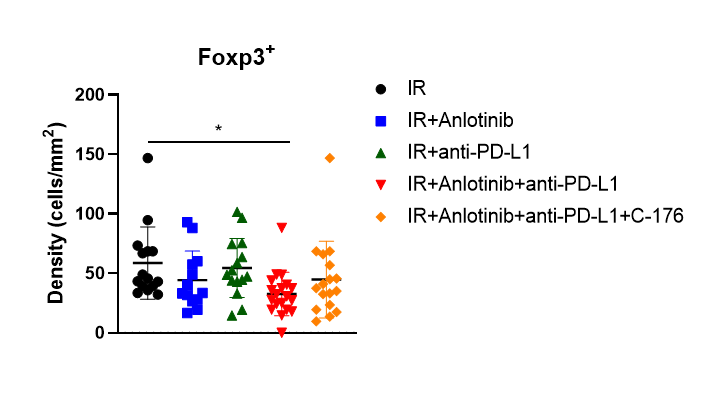

Supplement: Supplementary file 8 — Supplementary Figure 7 [file 41420_2022_1256_MOESM8_ESM.tif]

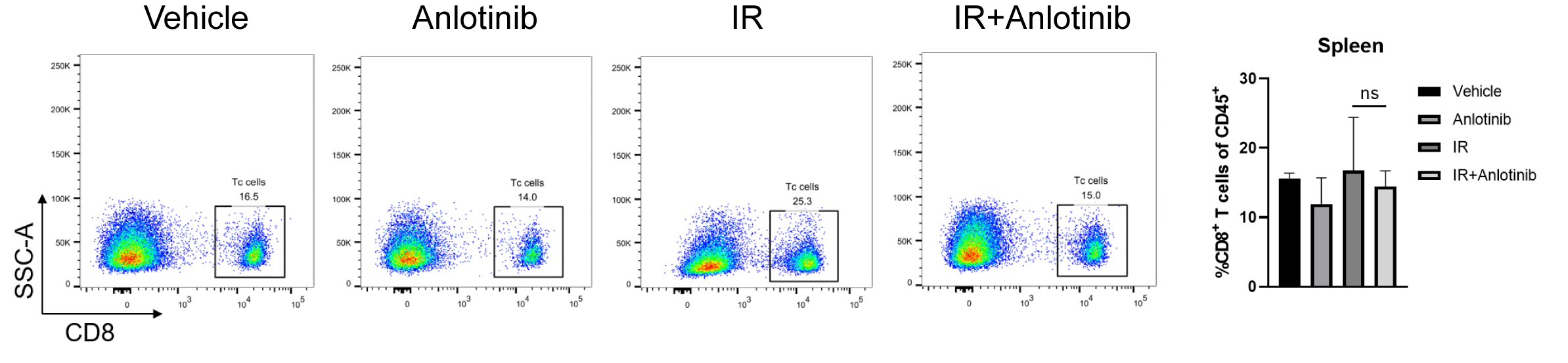

Supplement: Supplementary file 9 — Supplementary Figure 8 [file 41420_2022_1256_MOESM9_ESM.tif]

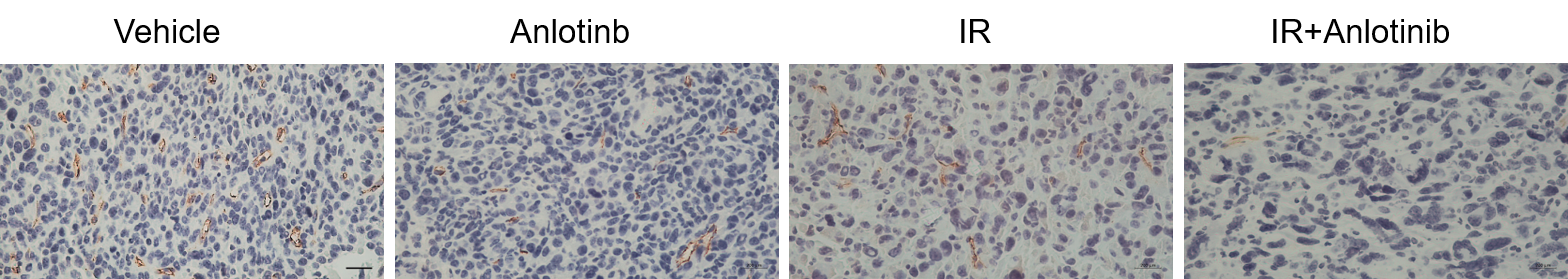

Supplement: Supplementary file 10 — Supplementary Figure 9 [file 41420_2022_1256_MOESM10_ESM.tif]

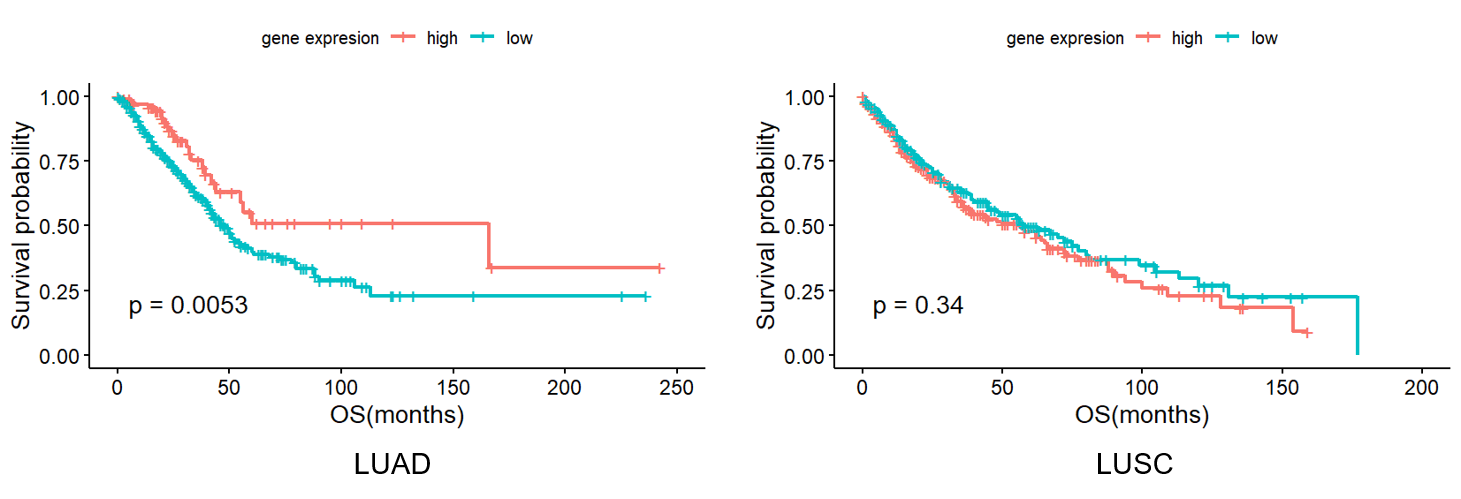

Supplement: Supplementary file 11 — Supplementary Figure 10 [file 41420_2022_1256_MOESM11_ESM.tif]

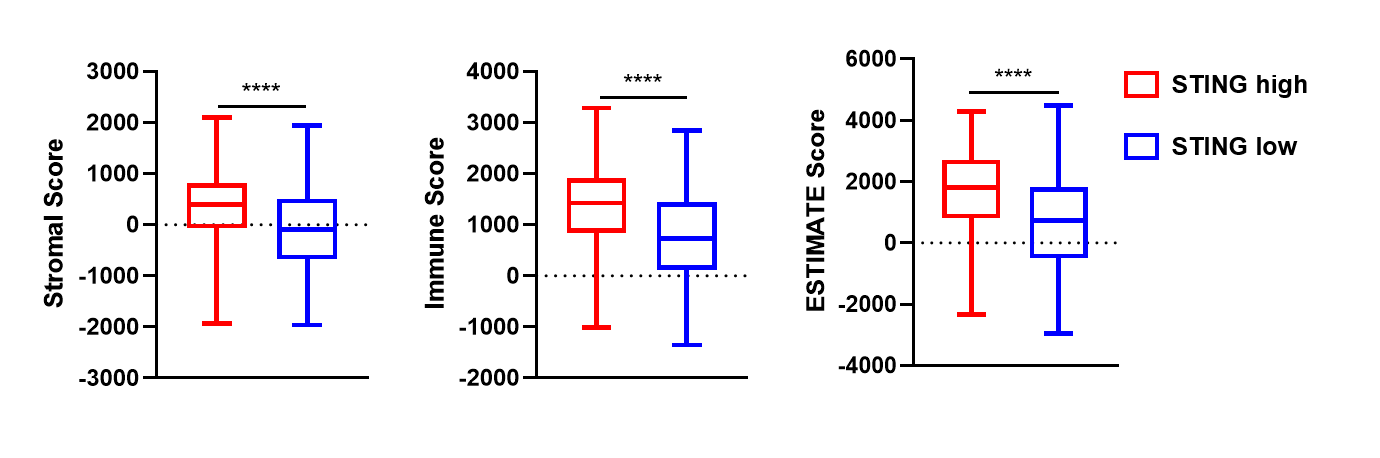

Supplement: Supplementary file 12 — Supplementary Figure 11 [file 41420_2022_1256_MOESM12_ESM.tif]

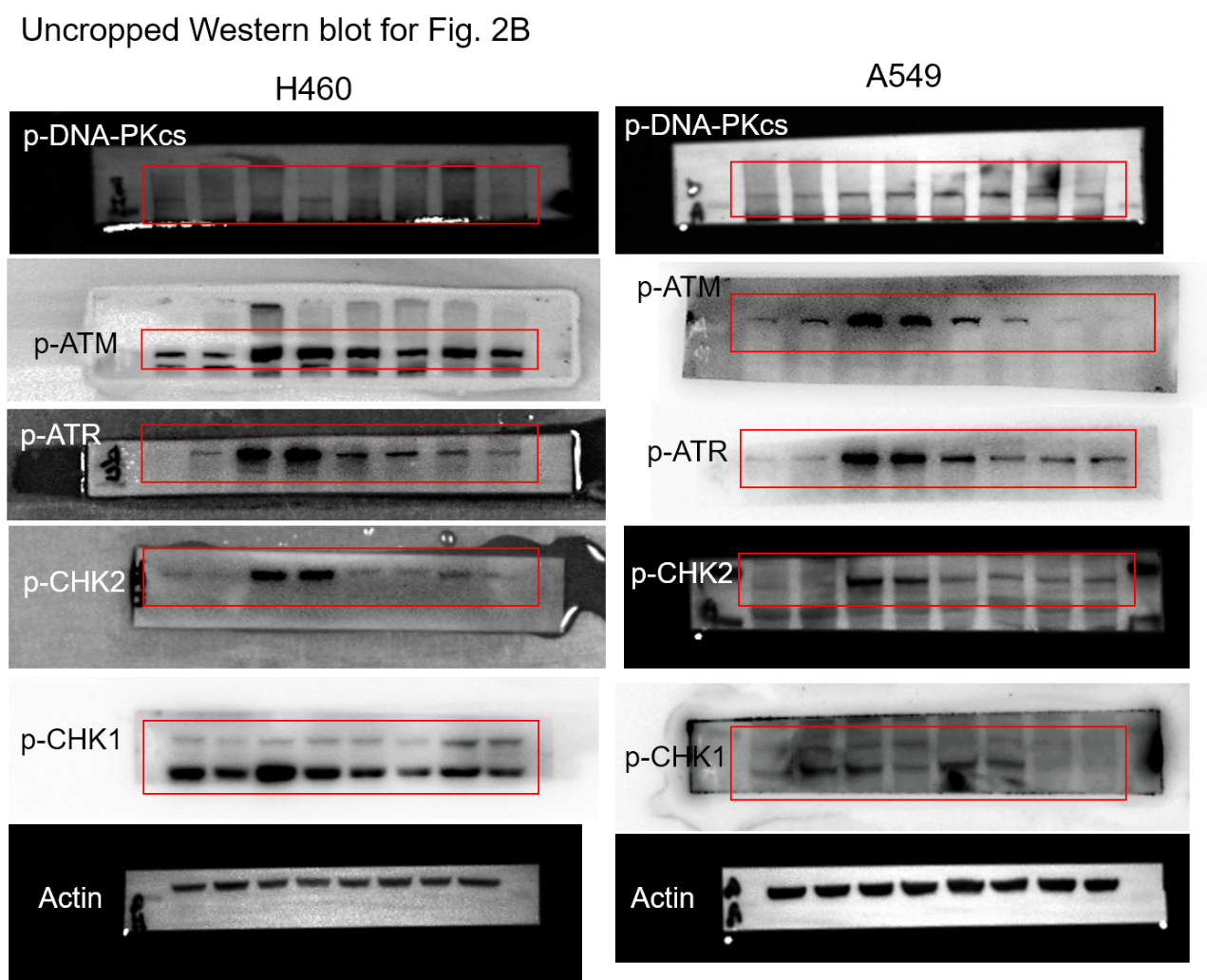


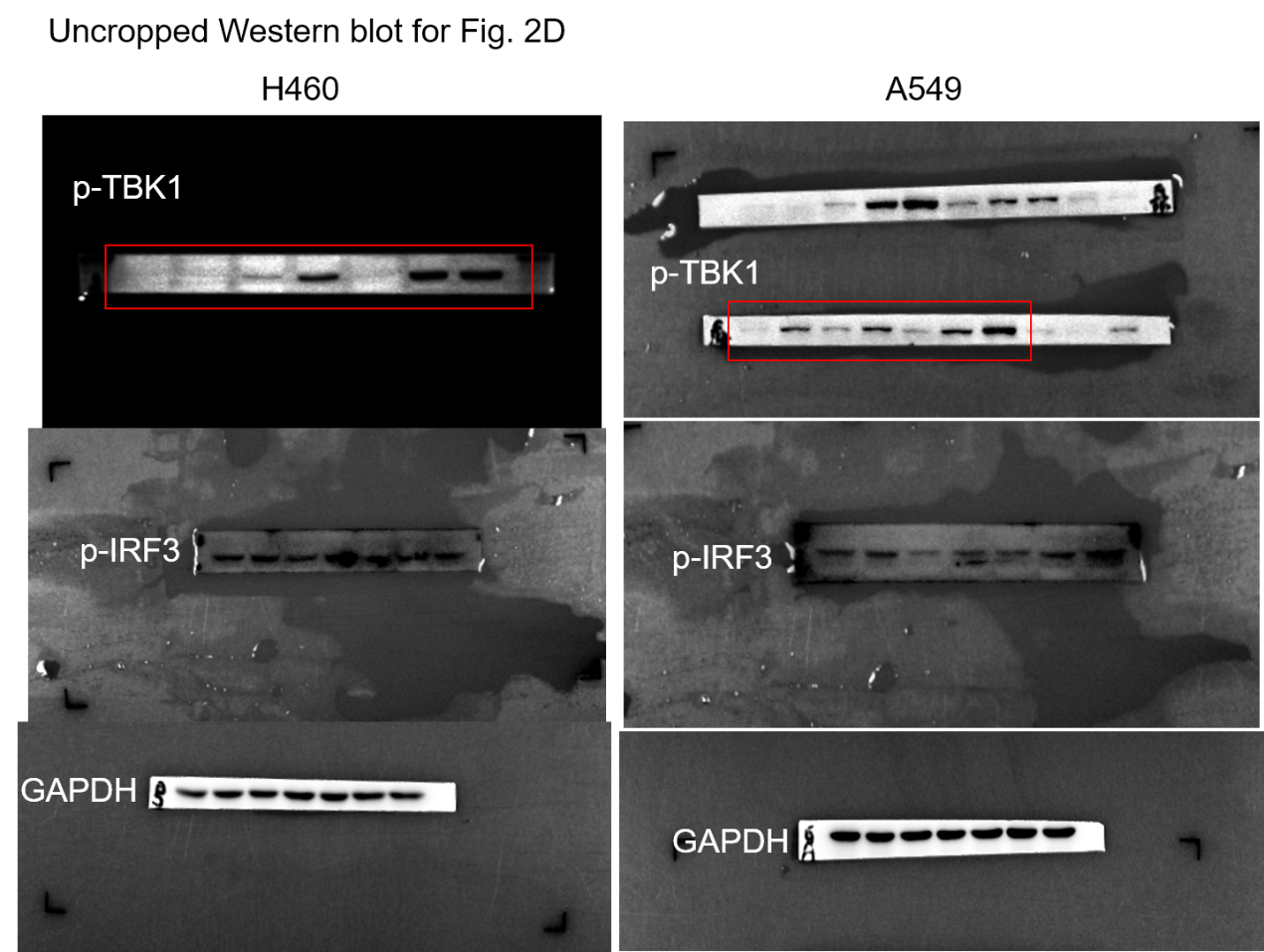


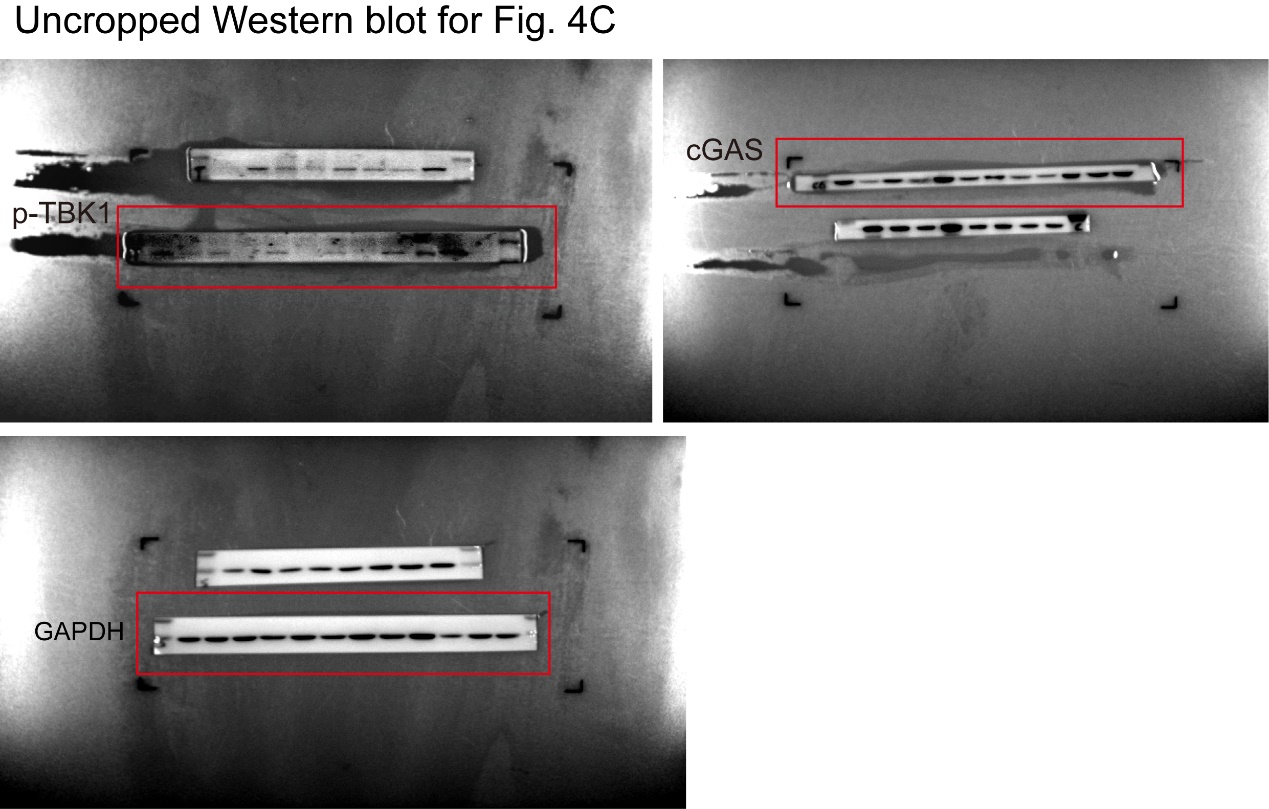


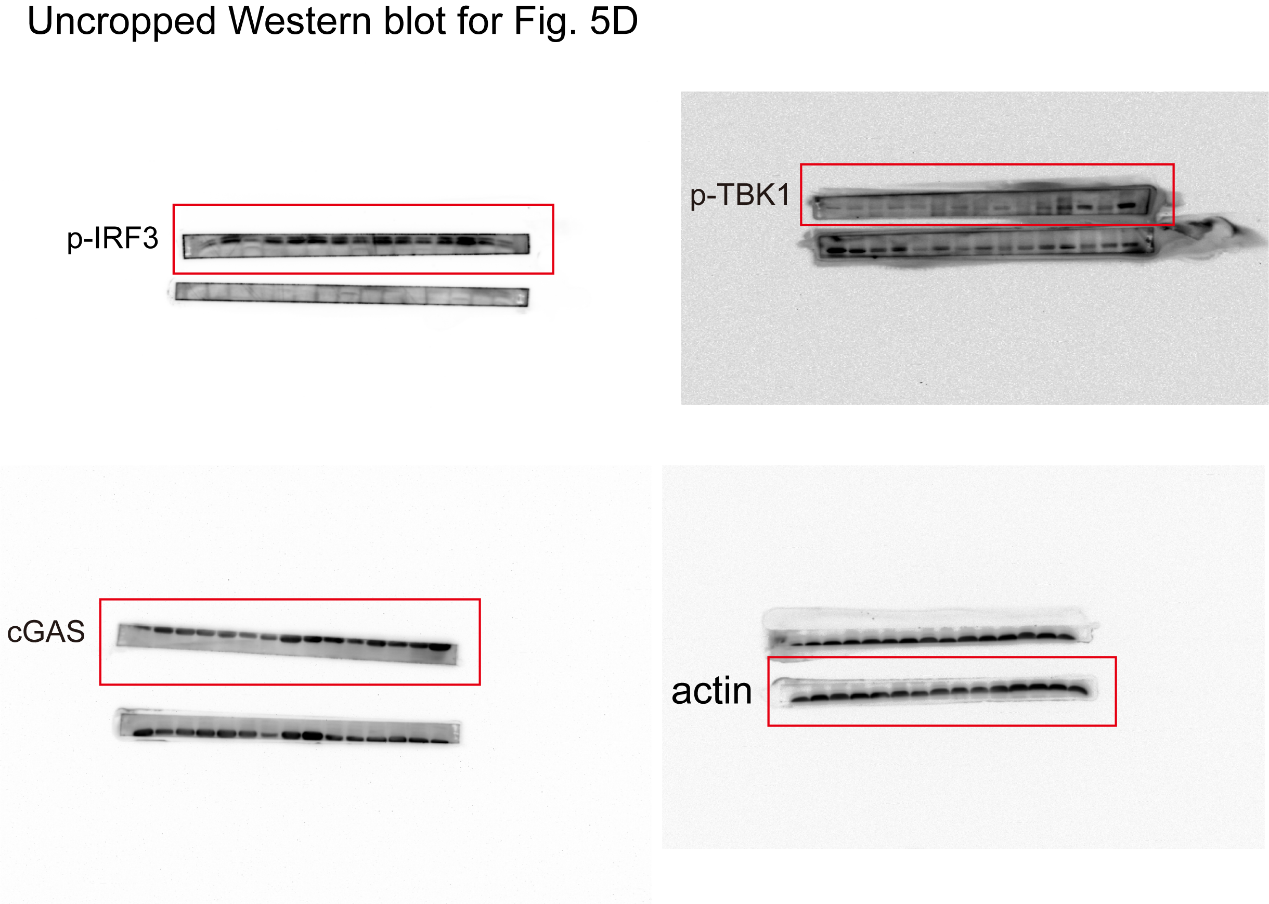


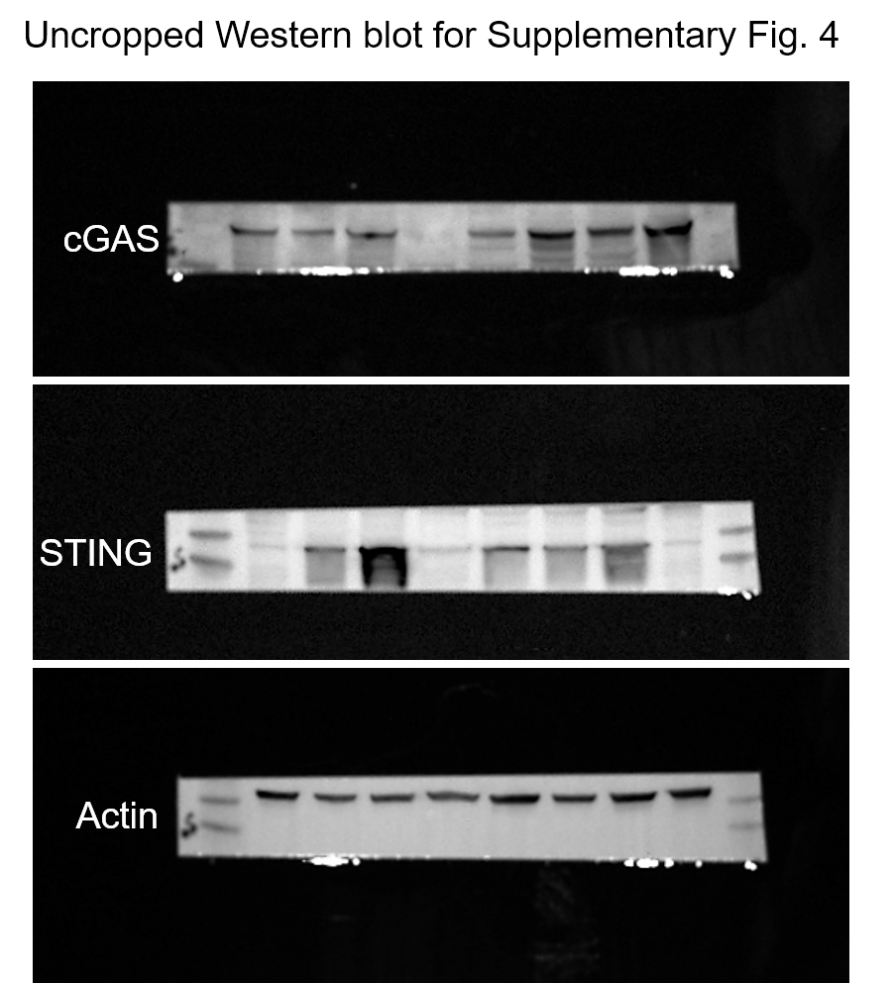

Supplement: Supplementary file 15 — Original Data File [file 41420_2022_1256_MOESM15_ESM.docx]
